# Transcriptomic and epigenomic remodeling occurs during vascular cambium periodicity in *Populus tomentosa*

**DOI:** 10.1038/s41438-021-00535-w

**Published:** 2021-05-01

**Authors:** Bo Chen, Huimin Xu, Yayu Guo, Paul Grünhofer, Lukas Schreiber, Jinxing Lin, Ruili Li

**Affiliations:** 1 Beijing Advanced Innovation Center for Tree Breeding by Molecular Design, Beijing Forestry University, Beijing, 100083 China; 2 College of Biological Sciences and Biotechnology, Beijing Forestry University, Beijing, 100083 China; 3 Institute of Tree Development and Genome Editing, Beijing Forestry University, Beijing, 100083 China; 4 College of Biological Sciences, China Agricultural University, Beijing, 100193 China; 5 Institute of Cellular and Molecular Botany, University of Bonn, Kirschallee 1, D-53115 Bonn, Germany

**Keywords:** Plant stem cell, Cell wall

## Abstract

Trees in temperate regions exhibit evident seasonal patterns, which play vital roles in their growth and development. The activity of cambial stem cells is the basis for regulating the quantity and quality of wood, which has received considerable attention. However, the underlying mechanisms of these processes have not been fully elucidated. Here we performed a comprehensive analysis of morphological observations, transcriptome profiles, the DNA methylome, and miRNAs of the cambium in *Populus tomentosa* during the transition from dormancy to activation. Anatomical analysis showed that the active cambial zone exhibited a significant increase in the width and number of cell layers compared with those of the dormant and reactivating cambium. Furthermore, we found that differentially expressed genes associated with vascular development were mainly involved in plant hormone signal transduction, cell division and expansion, and cell wall biosynthesis. In addition, we identified 235 known miRNAs and 125 novel miRNAs. Differentially expressed miRNAs and target genes showed stronger negative correlations than other miRNA/target pairs. Moreover, global methylation and transcription analysis revealed that CG gene body methylation was positively correlated with gene expression, whereas CHG exhibited the opposite trend in the downstream region. Most importantly, we observed that the number of CHH differentially methylated region (DMR) changes was the greatest during cambium periodicity. Intriguingly, the genes with hypomethylated CHH DMRs in the promoter were involved in plant hormone signal transduction, phenylpropanoid biosynthesis, and plant–pathogen interactions during vascular cambium development. These findings improve our systems-level understanding of the epigenomic diversity that exists in the annual growth cycle of trees.

## Introduction

Wood is one of the most important natural and renewable resources on earth and it is the major raw material for the production of paper. To modify wood quality, it is essential to understand the underlying regulatory mechanisms of wood formation, especially the biological process of secondary growth in xylophyta^1–3^. Secondary growth mainly depends on the development of the vascular cambium and cork cambium^4,5^. Vascular cambium activity, which plays a critical role in wood formation, is highly complex and dynamic, resulting in a cumulative increase in girth by generating secondary xylem inward and secondary phloem outward in the plant stem^6,7^. Furthermore, the activity of the vascular cambium exhibits evident annual periodicity, including the dormant, reactivating, and active stages^8,9^. Therefore, to further improve the properties of wood by molecular modification, it is essential to deeply understand the regulatory mechanisms of cambium activity.

DNA methylation is an important epigenetic modification that plays vital roles in the transcriptional regulation of genes^10,11^. In plants, DNA methyltransferases maintain DNA methylation by recognizing different methylation contexts (CG, CHG, and CHH, where H = A, C, or T)^12^. For example, CG methylation and CHG methylation were maintained by MET1 and CMT3, respectively. In addition, CMT2 acts in several CHH methylation contexts to different degrees^13,14^. The regulation of DNA methylation was reported to be essential for normal germination of seeds^15–17^ and fruit ripening^18^. Specifically, it has been shown that the maternal genome is hypomethylated in plants and this DNA hypomethylation is initiated in the central cell during plant sexual reproduction^19–21^. Interestingly, the dynamics of DNA methylation and demethylation can regulate many biotic and abiotic stresses^22–24^. More recently, distinct DNA methylation dynamics over transposable element (TE) sequences were uncovered during the early stages of plant development^25^. DNA methylation deficiency caused by DDM1 or MET1 mutations is enough to activate the transcription of demethylated TE sequences and transpose some of these activated TEs^26,27^.

Transcriptomic sequencing technology provides convenience for exploring the molecular mechanisms underlying the growth and development of woody plants^28,29^. Chano et al. explored the transcriptomic basis of the formation of traumatic wood in conifers^30^. In *Liriodendron tulipifera*, the regulatory mechanisms underlying petal coloration were revealed by transcriptomic profiling^31^. In recent years, transcriptional mechanisms underlying the activity–dormancy transitions have been elucidated in poplar^1,32,33^. Furthermore, expressed sequence tag analysis has expanded our knowledge of gene regulation in wood formation^34,35^. In particular, the genes involved in cambium development were gradually revealed, which improved our understanding of wood formation in woody plants^1,36^.

MicroRNAs (miRNAs) play important regulatory roles in gene expression at the posttranscriptional level during plant growth and development^37–39^. In poplar, they have been found to play critical roles in regulating diverse developmental processes^40–42^ and responding to stress conditions^43–45^. In addition, miR166 is believed to be associated with targeting class III HD-ZIP transcripts, which influence cambium initiation and vascular tissue development^46,47^. Interestingly, the expression of Pta-miR166 was much higher during winter dormancy, suggesting that it can regulate season responses and development in perennial plants^47^. miR156 and miR172, whose functions have been well verified in *Arabidopsis*^48^, have been found to be of great importance in regulating the phase transition in the vascular cambium^2,49^.

In the present study, we collected dormant cambium (DC), reactivating cambium (RC), and active cambium (AC) tissues of poplar using an accurate tangential cryosectioning method and performed comprehensive analysis of the DNA methylation, transcriptome, and miRNA profiles during vascular cambium development. Our results provide new insight into the dynamics and interactions of the epigenome and transcriptome during vascular cambium development. The results will facilitate further understanding of the regulatory mechanisms during the development of the vascular cambium in trees. In addition, this study will lay a foundation for further revealing the molecular network underlying wood formation.

## Results

### Morphological changes in the cambial zones during the annual growth cycle in poplar

**Fig. 1:**
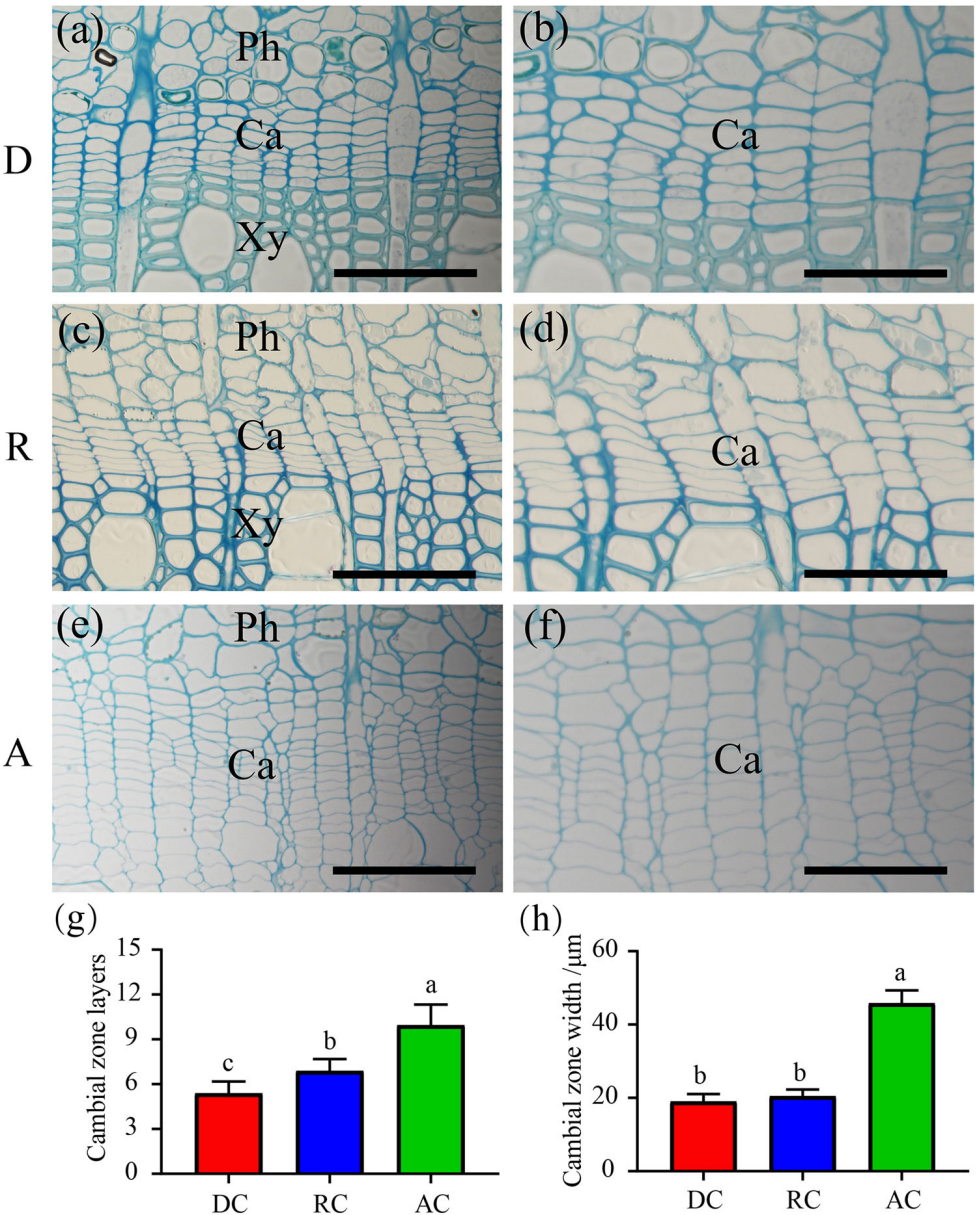
Anatomical observation of poplar cambial zones. Transverse sections showed the morphological features of dormant (**a**, **b**), reactivating (**c**, **d**), and active (**e**, **f**) cambial zones. The same magnifications (**b**, **d**, **f**) shown on the right. Significant differences are denoted by different letters (*P* < 0.05; one-way ANOVA followed by Tukey’s multiple range test). A: active, Ca: cambium, D: dormant, Ph: phloem, R: reactivating, Xy: xylem. Scale bars: 50 µm (**a**, **c**, **e**), 30 µm (**b**, **d**, **f**)

The vascular cambium consists of a group of cells located between the secondary phloem and xylem, which adds to the girth of the axis via the production of secondary phloem outside and xylem inside, respectively. It exhibits various morphological characteristics along with seasonal changes, including dormant, reactivating, and active periods. We originally conducted semi-thin sectioning to confirm the different stages, which can be used as a critical indicator during the dormant–active cycle. As shown in Fig. 1a, b, it was obvious that during the dormant stage, the cambial zone cells were compactly arranged and could be easily distinguished from the thickened secondary xylem cells and differentiated secondary phloem cells. Furthermore, the cambial zone at the active stage exhibited a significant increase in width (~46 µm) compared to that at the dormant stage (~19 µm), with loosely arranged cells (Fig. 1e, f). In the reactivating stages (Fig. 1c, d), the cambial zone possessed more cell layers (approximately seven layers) than that in the dormant stages (approximately five layers), but fewer layers than that in the active development stages (approximately ten layers) (Fig. 1g, h). The higher resolution of the cellular morphology of the vascular cambium allowed us to separate the cambium samples accurately for high-throughput sequencing.

### Dynamic changes in gene expression during cambium activity periodicity

To obtain more detailed and accurate information from the transcriptome, a total of nine transcriptome libraries were constructed from the dormant, reactivating, and active stages of the cambium for Illumina high-throughput sequencing. After all adapter sequences and low-quality reads were removed, a total of 72,611,012, 62,592,302, and 71,398,908 clean reads were acquired from the dormant, reactivating, and active stages, respectively (Supplementary Table S1). We globally profiled mRNA expression in DCs, RCs, and ACs. With the filter conditions of a fold change > 4 and false discovery rate (FDR) < 0.01, we obtained differentially expressed genes (DEGs) through pairwise comparisons. A clustering heatmap showed the expression patterns of the DEGs in the different samples (Supplementary Fig. S1a and Table S2). The Venn diagram indicates the number of DEGs specific to each comparison group and the number of DEGs common to each comparison group. A total of 8484 DEGs were found in the comparison between DC and AC conditions, accounting for the maximum. The numbers of DEGs retrieved from DC vs. RC and RC vs. AC were 6258 and 2690, respectively. Among all of these DEGs, 639 genes were differentially expressed in each comparison group (Supplementary Fig. S1b). Gene Ontology (GO) enrichment analysis indicated that the DEGs were associated with various biological processes involved in cambium activity periodicity, such as regulation of meristem growth, xylem development, cell division and expansion, cell wall biosynthesis, plant hormone signaling, and wood formation (Supplementary Figs. S1c and S2).

**Fig. 2:**
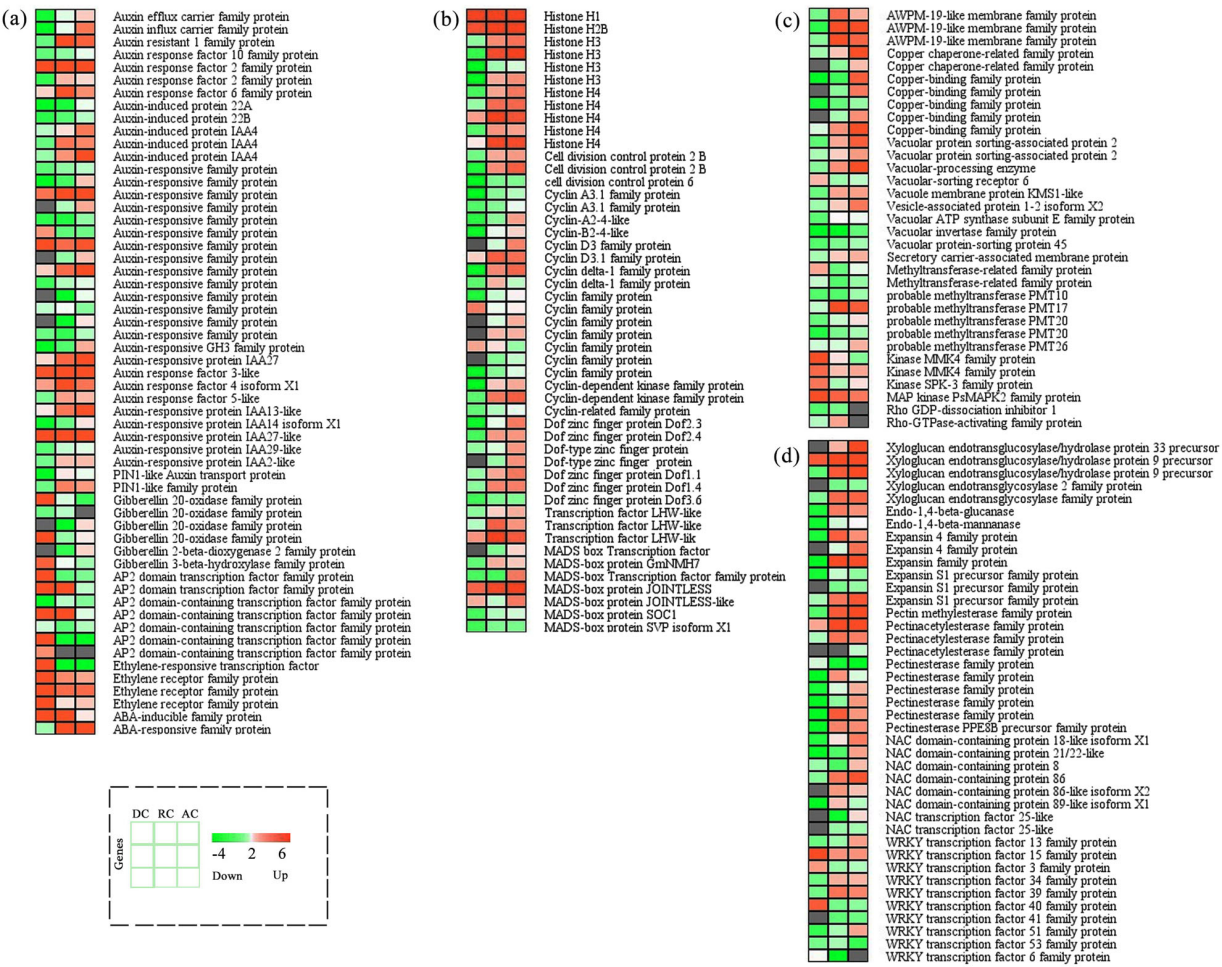
Expression pattern analysis of DEGs associated with different biological processes during cambium development in poplar. Heatmap showing the expression profile of DEGs involved in plant hormone signaling (**a**), cell division (**b**), copper-binding proteins (**c**), and cell expansion and cell wall biosynthesis (**d**). The bar represents the expression levels (the average FPKM (log_10_ scale)) of each gene in the dormant, reactivating, and active cambium. Red represents upregulation and green represents downregulation. AC: active cambium, DC: dormant cambium, FPKM: fragments per kilobase of exon per million fragments mapped, RC: reactivating cambium

To further investigate the dynamic changes and functions of the DEGs during cambium activity periodicity, we selected several DEGs belonging to different categories associated with the development of the vascular cambium (Supplementary Table S3). We found several DEGs coding proteins associated with plant hormones, such as auxin-associated DEGs (auxin-induced protein, auxin-responsive protein, auxin efflux/influx carrier family protein, PIN-FORMED-like auxin transport protein), gibberellin-associated proteins, ethylene-associated proteins (ethylene receptor and ethylene-responsive transcription factor), and abscisic acid-induced proteins (Fig. 2a). Cell division and cell expansion are essential events that occur during the development of cambium in wood formation and we also found several correlative DEGs. For instance, histones, cyclin family proteins, zinc finger proteins, and MAD-box transcription factors all exhibited highly dynamic changes (Fig. 2b). Furthermore, cell wall-associated proteins (endo-1,4-β-glucanase, pectin esterase/acetyl esterase, xyloglucan endotransglucosylase, NAC domain transcription factors, copper-binding proteins, and WRKY transcription factors) involved in the biosynthesis of pectin, lignin, and cellulose also showed various expression patterns during the dormant–active cycle (Fig. 2c, d). For example, several proteins related to cell wall biosynthesis and cell division exhibited higher expression in AC and RC than DC, consistent with the expectation for actively dividing cells. To validate these expression patterns of genes from the RNA sequencing (RNA-Seq) results, we randomly selected 18 DEGs for quantitative real-time (qRT)-PCR analysis. Experimental results proved that their expression patterns were consistent with their relative abundances from the transcriptome analysis (Supplementary Fig. S3).

### Identification and expression analysis of miRNAs during cambium activity periodicity

To identify small regulatory RNAs and the patterns of distribution during the dormancy–activity cycle in poplar, nine small RNA (sRNA) libraries from the dormant, reactivating, and active stages of the cambium with three biological replicates were constructed and subjected to next-generation sequencing. The length distribution of the sRNA showed a similar pattern in each library. The majority of the clean reads obtained from sequencing ranged from 20 to 24 nt (Supplementary Fig. S1d), with the major peak of each sample at 24 nt. The minor peaks differed, with the minor peaks of DC at 22 nt, RC at 21 nt, and AC at 23 nt. The peak at 24 nt represented the most abundant class of sRNA, consisting of 28.3% DCs, 25% RCs, and 38.4% ACs (Supplementary Table S4). A total of 360 miRNAs were identified after bioinformatics analysis, 235 of which are known and 125 were newly predicted.

**Fig. 3:**
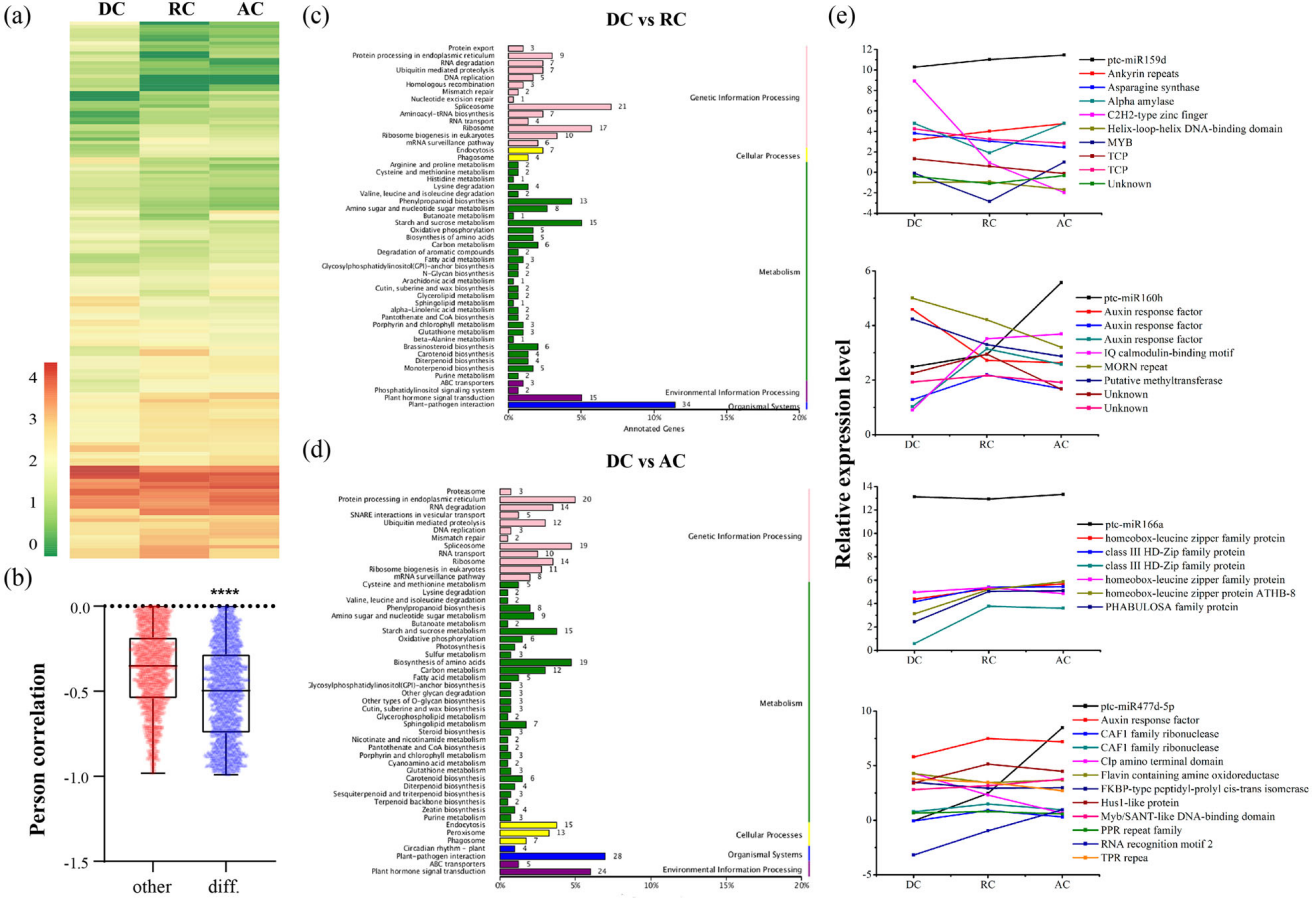
Expression patterns and functional annotations of differentially expressed miRNAs and their targets during cambium activity periodicity. **a** Heatmap showing the expression patterns of differentially expressed miRNAs during cambium activity periodicity. The relative expression levels were hierarchically clustered. **b** Boxplot showing the expression correlations of differentially expressed miRNA/target pairs (diff.) and other miRNA/target pairs. **c**, **d** KEGG pathway enrichment analysis for the target genes of differentially expressed miRNAs during comparison among DC vs. RC (**c**) and DC vs. AC (**d**). **e** Expression profiles of several important miRNAs (miRNA159d, miRNA160h, miRNA166a, and miRNA477d-5p) and their target genes. Significant differences are denoted by asterisks (****P < 0.0001; Student’s *t*-test) in **b**. AC: active cambium, DC: dormant cambium, RC: reactivating cambium

To explore the expression level changes and potential regulatory roles of these miRNAs, we calculated the expression levels of all of the miRNAs and analyzed the differentially expressed miRNAs by pairwise comparisons. The number of differentially expressed miRNAs between DC and RC was 44, which was less than that found for DC vs. AC (121) and RC vs. AC (127). Fourteen miRNAs exhibited different expression patterns during cambium activity periodicity (Supplementary Fig. S1e). The expression patterns of the differentially expressed miRNAs are shown in the cluster map (Fig. 3a and Supplementary Table S5). Based on the Pearson’s correlation coefficient calculated by R scripts, 3334 miRNA/target pairs displayed negative correlations, whereas 2928 pairs exhibited positive correlations (Supplementary Table S6). Furthermore, differentially expressed miRNA/target pairs exhibited stronger negative correlations than other miRNA/target pairs (Fig. 3b and Supplementary Table S7). Furthermore, pairwise comparisons were performed with the target genes of the differentially expressed miRNAs and the functional interpretation of these predicted target genes was investigated by Kyoto Encyclopedia of Genes and Genomes (KEGG) analysis. The results from DC vs. AC and DC vs. RC showed that these genes are mainly involved in certain pathways, such as plant hormone signal transduction, cell wall-associated pathways (phenylpropanoid biosynthesis and carbon metabolism), plant–pathogen interaction, and transcriptional regulation (Fig. 3b, c). Moreover, we selected several miRNAs involved in cambium development and analyzed the expression patterns of these miRNAs and predicted target genes. Interestingly, pto-miR159 and pto-miR477 were highly abundant in RC and increased during the transition from the reactivating stage to the active stage, whereas pto-miR166 showed the opposite, namely, the expression in RC was lower but elevated during the transition from the reactivating stage to the active stage. In addition, pto-miR160, which was reported to be involved in auxin signal transduction, was expressed specifically during dormancy release, with a twofold or higher relative expression level in AC than in RC and DC. Finally, we analyzed and predicted the target genes of these vital miRNAs (Fig. 3d). For instance, class III HD-ZIP family proteins, as the target genes of pto-miR166, exhibited higher expression levels in RC and AC, and thus may be involved in cambium initiation and vascular tissue development. To validate these expression patterns of miRNAs from the miRNA profiles, we randomly selected six differentially expressed miRNAs for qRT-PCR analysis. The results implied that their expression patterns were in accordance with their relative abundances by miRNA profiles (Supplementary Fig. S4).

### Global DNA methylation changes occur during cambium activity periodicity

**Fig. 4:**
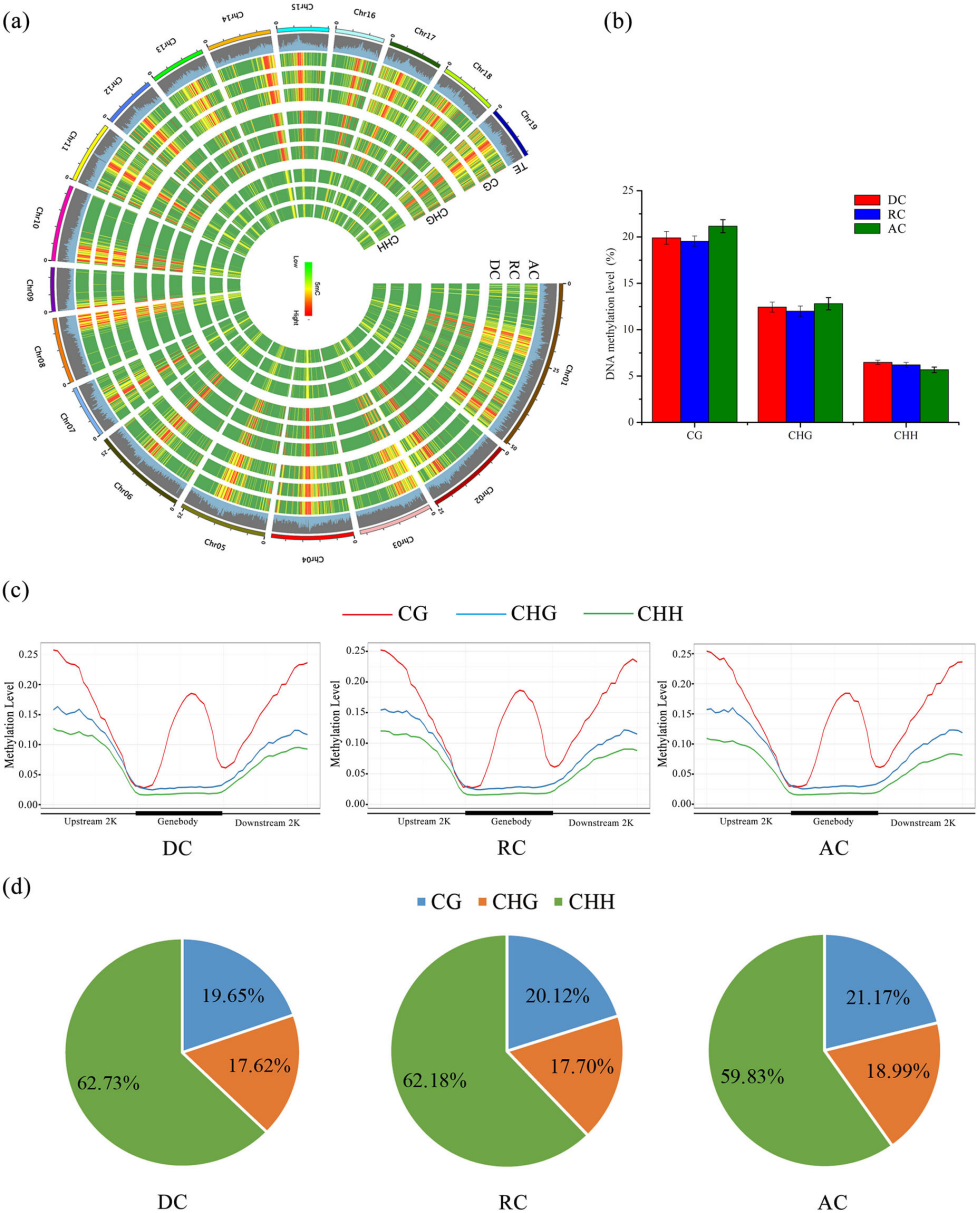
Methylome and genome architecture during cambium activity periodicity in poplar. Bisulfite sequencing data from each sample were merged and used to call the methylated regions. **a** Epigenome density plot of DNA methylation in the dormant, reactivating, and active cambium. Methylation density is represented in 100 kb blocks separated by different methylation contexts and samples. Red represents a high methylation level and green represents a lower methylation level. The outer annotation track represents the position of transposons (TEs). **b** Histogram showing the genome-wide average methylation levels for each methylation context in the dormant cambium, reactivating cambium, and active cambium. **c** DNA methylation patterns of DCs, RCs, and ACs in different genomic regions for each methylation context were calculated by the fraction method. **d** Relative percentage of methylated cytosines in the three contexts (CG, CHG, and CHH) in poplar. AC: active cambium, DC: dormant cambium, RC: reactivating cambium

To further reveal the dynamics of DNA methylation variation throughout the cambium activity period, direct comparisons were performed to identify whether stage-specific differences in DNA methylation existed during cambium activity periodicity. The data from whole-genome bisulfite sequencing and the conversion rates of each sample are shown in Supplementary Table S8. The distribution of cytosine methylation was described at the chromosome level, and a poplar epigenome density plot of DC, RC, and AC for the three methylation types (CG, CHG, and CHH) at the genome-wide chromosome level was illustrated in a Circos diagram. In some instances, CG, CHG, and CHH methylation was prevalent near the centromere, such as on chromosomes 4, 5, and 6, but was far from the centromere on other chromosomes (Fig. 4a). Global methylation analysis revealed that CG and CHG were the most stable types throughout the cambium activity periodicity in poplar (Fig. 4b). The average genome-wide methylation levels for CG, CHG, and CHH methylation contexts were similar during the three stages of the vascular cambium. Nevertheless, there were still some slight changes in each methylation context, with higher methylation levels of CG and CHG, and a slightly lower level of CHH in the AC (Fig. 4b).

We also analyzed the DNA methylation patterns of protein-coding genes across different genomic regions, and the results indicated that these patterns were largely similar during cambium activity periodicity using different calculation methods (Fig. 4c and Supplementary Fig. S5). The CG methylation context was the highest among the three methylation contexts, with CHH exhibiting the lowest methylation level. CG methylation showed the highest coverage in the upstream and downstream 2k regions, with significant decreases at the 5′ and 3′ borders of the gene body. In the central part of the gene body, an obvious peak in CG methylation appeared. The levels of CHH and CHG methylation were lowest in the gene body region relative to the 5′ and 3′ flanking regions as previously described, and the CHH methylation level was persistently lower than that of CHG across the genomic regions (Fig. 4c). Furthermore, to analyze the distribution of methylcytosine sites in the different methylation contexts during cambium development, the proportion of CG, CHG, and CHH on the methylcytosine sites was counted (Supplementary Table S9). As shown in Fig. 4d, the proportions were similar during cambium activity periodicity. The most frequent occurrence was found at CHH sites (~60%), with less frequency in the CG and CHG contexts (~20% and 18%, respectively). Compared with those of DC and RC, the ratios of CG and CHG were slightly increased, and a decreased proportion of CHH was obtained in the AC.

### Correlation between gene expression levels and DNA methylation

To explore the potential regulatory functions of the promoter, gene body, and downstream 2 kb methylation on gene expression, DNA methylome analysis was performed with DC, RC, and AC using the same materials as the transcriptome analysis. According to the expression level, all the genes were divided into four groups, among which the first represented the highest expression level and the fourth represented the lowest. As shown in Supplementary Fig. S6, along most of the gene body, most of the highly expressed genes showed high CG methylation levels. However, relatively low CG methylation in the promoter (upstream 2 kb) region and downstream 2 kb region was found (Supplementary Fig. S6, left). Interestingly, CHG and CHH methylation levels showed a negative correlation with expression mostly in the downstream region (Supplementary Fig. S6, middle and right).

**Fig. 5:**
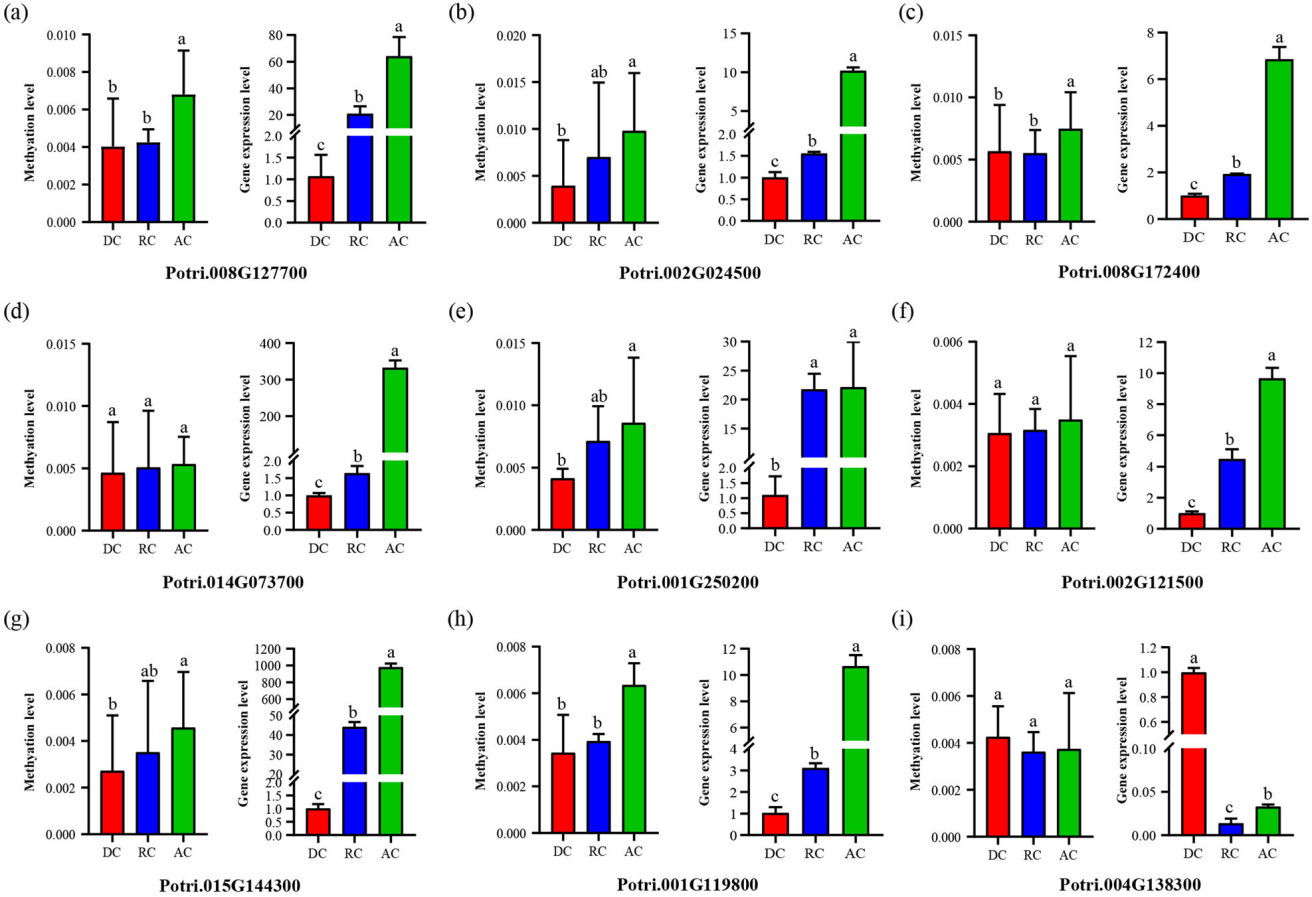
Correlation between gene expression levels and DNA methylation in the gene body. **a** Potri.008G127700, encoding an auxin efflux carrier family protein. **b** Potri.002G024500, encoding an auxin-responsive family protein. **c** Potri.008G172400, encoding an IAA13-like protein. **d** Potri.014G073700, encoding a gibberellin 20-oxidase family protein. **e** Potri.001G250200, encoding a cell division control protein 6. **f** Potri.002G121500, encoding a cyclin A3.1 family protein. **g** Potri.015G144300, encoding a copper-binding family protein. **h** Potri.001G119800, encoding a vacuolar-processing enzyme. **i** Potri.004G138300, encoding a kinase SPK-3 family protein. In each subfigure, the left histogram represents the gene body methylation levels and the right histogram represents the gene expression levels validated by qRT-PCR analysis. Experiments were performed with three biological replicates × three technical replicates. Significant differences are denoted by different letters (*P* < 0.05; one-way ANOVA followed by Tukey’s multiple range test). AC: active cambium, DC: dormant cambium, RC: reactivating cambium

To further explore the correlation between DNA methylation and gene expression patterns, we selected several DEGs involved in multiple vital biological processes during cambium development that were only methylated in the gene body region and validated their expression levels by qRT-PCR analysis. As shown in Fig. 5, the gene body methylation level of these DEGs, including those involved in plant hormone signaling, cell division, and cell wall biosynthesis, was generally in accordance with the gene expression levels. For example, the methylation and gene expression levels of the auxin efflux carrier family protein Potri.008G127700 were both increased continually during cambium development (Fig. 5a). Interestingly, the gene body methylation level of Potri.001G250200, which encodes a cell division control protein, was increased gradually during annual periodicity, and the gene expression level of Potri.001G250200 in RC and AC was also obviously elevated compared with that in DC (Fig. 5e). These results indicated that genes with only methylated gene bodies seemed to have a positive relationship with gene expression levels during cambium activity periodicity.

### Widespread dynamic differences in DMRs during cambium development

**Fig. 6:**
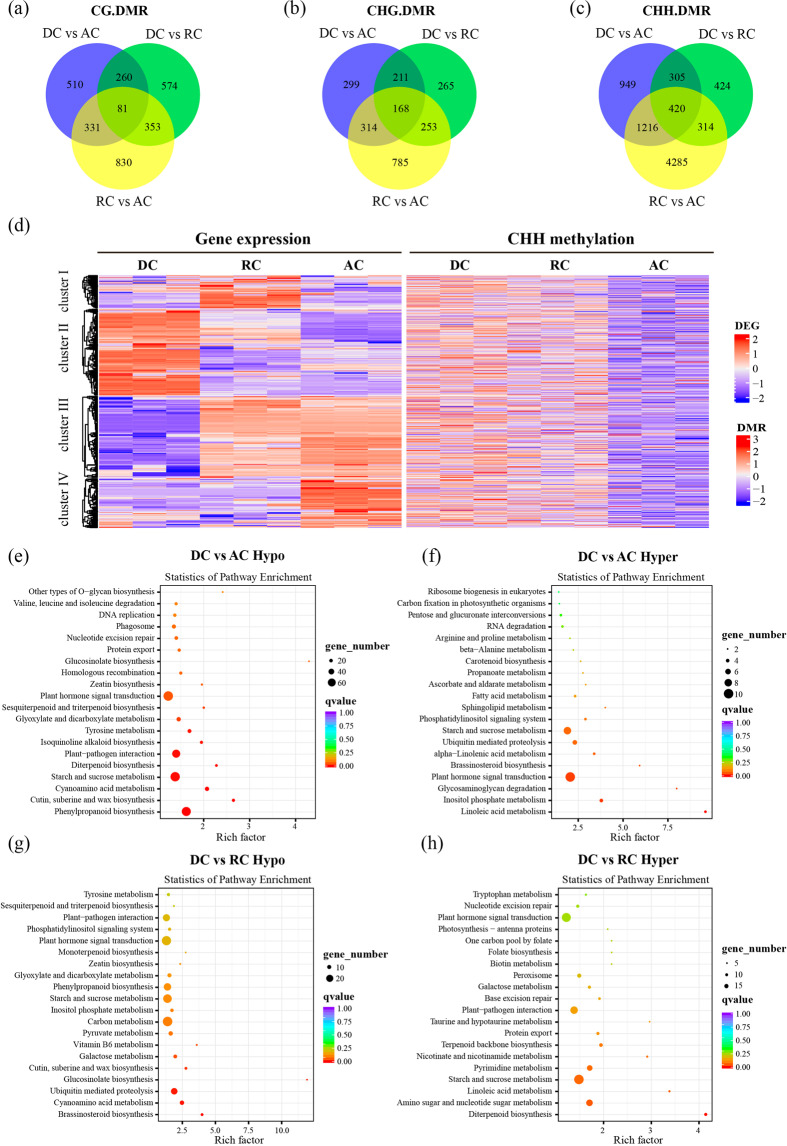
Differential methylome analysis among dormant, reactivating, and active vascular cambium. **a**–**c** Venn diagram of the number of DMR-associated genes during pairwise comparisons among dormant, reactivating, and active cambium. **d** Heatmaps showing the correlation between gene expression and CHH methylation in the promoter region. **e**–**h** KEGG analysis of hypermethylated and hypomethylated genes during comparison between DC vs. AC (**e**, **f**) and DC vs. RC (**g**, **h**). The size of the circle indicates gene numbers and the color indicates the *q*-value. AC: active cambium, DC: dormant cambium, DMRs: differentially methylated regions, RC: reactivating cambium

Differentially methylated regions (DMRs) between samples reflect the differences in regional methylation levels and DMRs in the promoter region cause changes in gene expression levels. For this reason, we investigated the number of DMRs of CG/CHG/CHH and annotated the DMRs on the basis of their location in the genome. We further analyzed the number of DMR-associated genes related to cambium activity periodicity. In total, we identified 1182 DMR-associated genes of CG in DC vs. AC, and 1268 and 1595 DMR-associated genes of CG in DC vs. RC and RC vs. AC, respectively (Fig. 6a). In addition, 992/2890 DMR-associated genes of CHG/CHH in DC vs. AC, and 897/1463 and 1520/6235 DMR-associated genes of CHG/CHH in DC vs. RC and RC vs. AC, respectively, were also identified (Fig. 6b, c). Most importantly, 81, 168, and 420 DMR-associated genes of CG, CHG, and CHH were all detected within the 3 periods of cambium development. The heatmaps also presented a widespread methylome change in DMRs during cambium activity periodicity (Supplementary Fig. S7). Interestingly, striking features included extensive hyper CHH for the RC and hypo CHH for the DC (Supplementary Fig. S7).

We further analyzed the distribution of hypermethylated and hypomethylated DMRs of CG/CHG/CHH across the genomic regions, which were divided into promoter, first exon, gene body, and intergenic regions. In total, 2234 hypermethylated and 12,602 hypomethylated DMRs were identified in DC vs. AC. Similarly, 3488 hyper-DMRs and 4820 hypo-DMRs were identified in DC vs. RC (Supplementary Table S10). The results from DC vs. RC showed that most hypermethylated and hypomethylated DMRs occurred at the intergenic region, with CHH methylation as the most predominant type. Similar results were also observed in the promoter region, whereas the distribution pattern in the gene body region was the reverse, with CG and CHG methylation being predominant (Supplementary Fig. S8a). We also observed that most of the hypomethylated DMRs were distributed in intergenic regions and CHH DMRs made up the largest percentage of the total DMRs from DC vs. AC and RC vs. AC (Supplementary Fig. S8b, c). By analyzing the relationship of DMRs and gene expression changes, we found that the gene expression and CHH methylation levels in the promoter regions displayed higher correlations than those in non-CHH-DMR overlapping genes (Supplementary Fig. S9). The expression patterns of these genes in the three stages (DC, RC, and AC) were divided into four clusters based on the *k*-means method (Fig. 6d). Gene expression in cluster I showed no significant correlation with DNA methylation levels. Genes in cluster II were downregulated from the DC to AC stage as the CHH methylation level decreased in the promoter regions. Furthermore, genes in clusters III–IV were upregulated from the DC to AC stage, accompanied by CHH hypomethylation in the promoter (Fig. 6d). It is worth noting that these genes were involved in plant hormone signal transduction, starch and sucrose metabolism, phenylpropanoid biosynthesis, and plant–pathogen interactions.

Moreover, we carried out KEGG pathway enrichment analysis to further study the biological functions of DMR genes. As shown in Fig. 6f, the hypermethylated genes in DC vs. AC were mainly assigned to pathways involved in plant hormone signal transduction, starch and sucrose metabolism, etc. The first two pathways were also enriched in DC vs. RC (Fig. 6h). Among the hypomethylated genes in DC vs. AC, the pathways of plant hormone signal transduction, starch and sucrose metabolism, phenylpropanoid biosynthesis, and plant–pathogen interaction were enriched (Fig. 6e). However, hypomethylated genes in DC vs. RC showed abundant enrichment in the carbon metabolism and ubiquitin-mediated proteolysis pathways (Fig. 6g). Functional annotation of hyper- and hypomethylated genes from RC vs. AC by KEGG analysis was also performed (Supplementary Fig. S10).

## Discussion

Plant growth and developmental processes have established complex adaptive mechanisms in response to various environmental changes. Vascular cambium development during the dormant, reactivating, and active stages involves various biological events, such as cambial stem cell maintenance, cell division, biosynthesis of the cell wall, and phytohormone biosynthesis. Auxin, an important hormone, plays a critical role in cambial stem cell activity by regulating the expression levels of auxin-associated factors, which is of great importance in the dormant–active transition^50,51^. In our investigation, we found that genes encoding auxin-induced proteins and auxin response factors (e.g., ARF 3-like) exhibited higher expression levels in RC and AC than in DC, indicating that the expression of the gene promotes the development of the cambium. This was also reported during the active–dormant transition in hybrid aspen^52^. During cambium activity from the dormant to active stage, the expression levels of genes associated with cell division (histone H4, cyclin) and cell wall synthesis (xyloglucan endotransglycosylase and pectin methylesterase) in RC and AC were also elevated in this study, which is consistent with active division and cell wall expansion^2,32^.

More recent studies have indicated that miRNAs in poplar have critical roles in development^49^ and the response to environmental stress^43,45,53^. For instance, miRNA 164 and miRNA 396 have been reported to be involved in the endodormancy release process^8^, and miRNA 159 and miRNA166 were reported to be involved in the response to cold and pathogen stress^54,55^, respectively. In our study, the correlations of differentially expressed miRNAs and target genes were stronger than those of other miRNA/target pairs (Fig. 3b), implying that differentially expressed miRNAs might play vital roles during cambium activity periodicity. As an auxin-related miRNA, miRNA160 plays an important role in the transition from endodormancy to ecodormancy^8^. In our study, the abundance of miRNA160 was increased continuously in the cambium of poplar during the dormant–active cycle. In contrast, its target genes, auxin response factor family proteins, decreased during the dormancy–reactivation transition. Therefore, it is reasonable to speculate that miRNA160, as a stage-specific regulator, plays a vital role in dormancy release. In addition, we detected the expression patterns of miRNA166 and its target gene during the dormant–active cycle, and the results showed that the expression of miRNA166 decreased slightly during the dormancy–reactivation transition. However, the expression of its predicted target genes, class III HD-ZIP family proteins of transcription factors, which have been reported to function during vascular development and wood formation^56^, increased from the dormant to reactivating stages, indicating the vital role of miR166 during cambium activity periodicity.

DNA methylation is a steady but reversible epigenetic modification that may play critical roles in regulating gene expression and controlling genome stability^57,58^. The relative methylation levels of CG, CHG, and CHH were first established in *Arabidopsis* by bisulfite sequencing^59,60^ and further studied in multiple species. During bud break in apple, genomic DNA methylation was found to be reduced, which further affected gene expression in biological processes^61^. Recently, Turco et al.^62^ identified DEGs involved in DNA methylation between vascular and nonvascular tissues, indicating that DNA methylation changes were a feature of root vascularization in sorghum. In our study, we showed that the CG methylation level in the cambium was 19.5–21.2%, that of CHG was 12.0–12.8%, and that of CHH was 5.7–6.5%, which was much lower than that in the flowers^42^ and leaves of poplar^63^, indicating the diversity of methylation patterns in different tissues of poplar. Moreover, we found that the CHH context harbors the largest proportion of all mCs during cambium activity from the dormant to active stage (Fig. 4d). Recent studies have also shown that CHH methylation varies significantly during biological processes^64,65^. In addition, in the course of analyzing the DMRs in the three comparison groups (DC vs. AC, RC vs. AC, and DC vs. RC), we observed that the number of CHH-DMR changes was the greatest during vascular cambium development (Supplementary Fig. S7). These results suggested that the changes in DNA methylation were mostly contributed by CHH methylation and the dynamic regulation of CHH methylation played an important role in the integrity and stability of the genome in *Populus tomentosa*. This is indeed supported by recent studies focusing on somatic embryogenesis of longan and soybean^66,67^. Furthermore, there was no monotonically increasing or decreasing trend with regard to the expression levels of genes associated with different DNA methylation pathways (Supplementary Fig. S11).

Epigenetic changes are more flexible than genetic variations, causing plants to more easily acclimate to various environments through epigenetic alterations^68^. A recent study on water deficit in apple by KEGG enrichment of DMR genes showed a common pathway in ubiquitin-mediated proteolysis, indicating that water deficit reformed the methylation status of genes associated with ubiquitin-mediated proteolysis^68^. Here we performed KEGG enrichment analysis of DMR genes between DC, RC, and AC, and the results indicated CHH-type DMR gene enrichment in starch and sucrose metabolism, plant hormone signal transduction, and phenylpropanoid biosynthesis, implying epigenetic alterations during vascular development. DNA methylation is known to repress gene expression, but additional evidence has revealed that the correlation between methylation status and gene expression seems to be more nuanced than previously thought^68,69^. The relationship between CG gene body methylation and gene expression has been studied in *Arabidopsis*, rice, and poplar^63,70,71^. In our study, we found that the highly expressed genes exhibited high CG methylation levels in the gene body and the positive relationship was further verified by qRT-PCR (Fig. 5). In contrast, CHG methylation levels showed a negative correlation mostly downstream (Supplementary Fig. S6, middle), consistent with a previous study in *cmt3* mutants^72^. Moreover, CHH methylation levels exhibited a positive relationship in the upstream and downstream regions in a study of maize^73^, whereas the results in our study showed that CHH methylation levels were negatively correlated with gene expression in the downstream region. Most importantly, we found that the expression of genes with CHH DMRs in the promoter displayed higher correlations with the DNA methylation levels than that of non-CHH-DMR genes (Fig. 6d and Supplementary Fig. S9). Strikingly, these genes were involved in plant hormone signal transduction, starch and sucrose metabolism, phenylpropanoid biosynthesis, and plant–pathogen interactions, implying the important role of CHH methylation during cambium periodicity in poplar.

## Conclusions

**Fig. 7:**
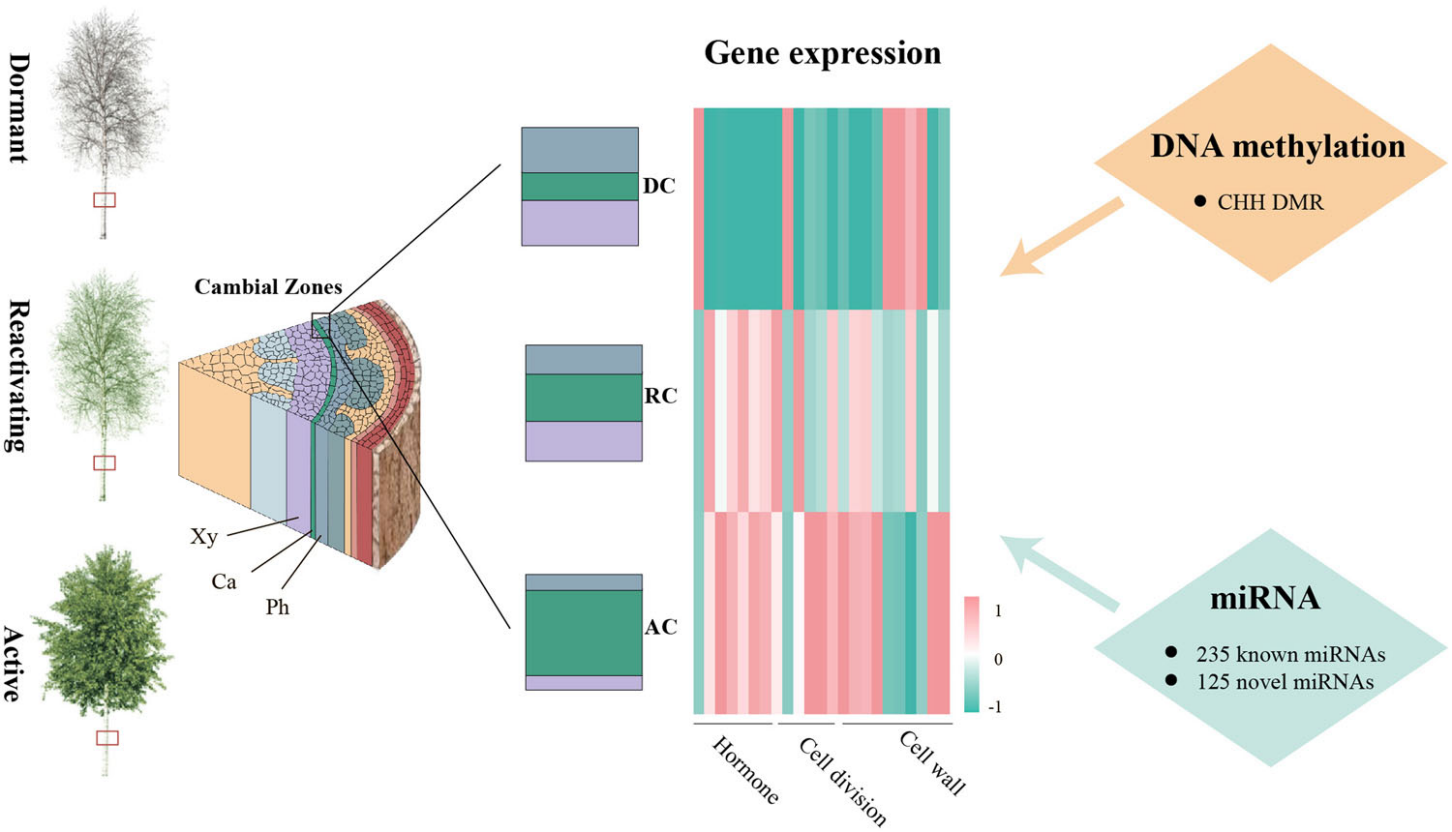
Schematic diagram showing the regulation of multiple genes, miRNAs, and DNA methylation during the annual growth cycle in the poplar vascular cambium. AC: active cambium, Ca: cambium, DC: dormant cambium, Ph: phloem, RC: reactivating cambium, Xy: xylem

The vascular cambium plays critical roles in wood formation as a result of the growth and differentiation of secondary phloem and secondary xylem. Our study provides the first description of epigenomic differentiation during seasonal developmental variation of the secondary meristem in a perennial plant at genome-scale resolution. The results present several of the predicted DEGs altering vascular cambium activity, as well as 235 known and 125 novel miRNAs. In addition, DMR analysis showed that hypomethylation mainly occurred in CHH contexts from DC to AC. Most importantly, we showed that the genes with hypomethylated CHH DMRs in the promoter were involved in plant hormone signal transduction, starch and sucrose metabolism, phenylpropanoid biosynthesis, and plant–pathogen interactions during vascular cambium development. Taken together, the regulation of cambium activity periodicity at various levels, including transcriptional, miRNA, and DNA methylation regulation, contributes to the adaptation to various growth and environmental changes, as shown in Fig. 7. This research provides new insight into the regulatory mechanism of vascular cambium development, which will potentially contribute to improved biomass production.

## Materials and methods

### Plant material and growth conditions

Poplar (*P. tomentosa* Carr) tree clones were grown under natural conditions in Guanxian County, a practice base of Beijing Forestry University located in Shandong Province, China. Different cambium materials were collected from the clones of LuMao 50 (~4 years old). The method of sampling was described previously^2^. Briefly, at each sampling time point, small wood blocks containing phloem, vascular cambium, and xylem were collected from three independent trees near breast height, almost 1.3 m above the ground. The cambium materials were collected on 21 January 2017, 30 March 2017, and 1 July 2017, corresponding to the dormant, reactivating, and active stages. The blocks were immediately placed into liquid nitrogen and then stored at −80 °C until use. The separation of the vascular cambium materials from the blocks was described previously^2^. Briefly, after being divided into small blocks, the vascular cambium materials separated by tangential cryosectioning using a cryomicrotome and Leica CM1850 Cryostat (Leica Microsystems Nussloch GmbH, Germany) were collected into 1.5 mL RNase-free tubes for DNA and RNA extraction.

### Morphological observations of the cambium zone

Samples of dormant, reactivating, and active stages of vascular cambium 4 mm^3^ in size and collected at breast height (1.3 m) were fixed with 2.5% glutaraldehyde in 100 mM phosphate buffer (pH 7.2) and included the secondary phloem, vascular cambium, and secondary xylem. After serial dehydration with ethanol and acetone, the samples were embedded in Spurr’s resin. A Leica microtome was used to obtain sections (1 μm). The sections were then stained with 0.25% (w/v) toluidine blue O (Sigma) and observed using an Olympus CX31 microscope equipped with a computer-assisted digital camera.

### DNA and RNA extraction

For the analysis of the methylome, miRNA profiles, and transcriptome, high-quality genomic DNA, miRNA, and total RNA were prepared. The genomic DNA of dormant, reactivating, and active vascular cambium meristem materials for whole-genome bisulfite sequencing was isolated using a Plant Rapid Genomic DNA Kit (Biomed, Beijing) according to the manufacturer’s instructions. The miRNA of the samples from the three stages for sRNA-Seq was extracted with an RN40-EASYspin plant microRNA kit (Aidlab, China) following the supplied protocol. Total RNA of the samples from the three stages for RNA-Seq was isolated using Plant RNA Purification Reagent (Invitrogen, USA) with an RNAprep Pure Plant Kit (TianGen, China) according to our improved method. A Nanodrop 2000 spectrophotometer and Agilent 2100 bioanalyzer were used to monitor RNA quantity and quality (OD260/280 ≥ 1.8; OD260/230 ≥ 1.0; RNA Integrity Number ≥ 8.0; 28 S/18 S ≥ 1.5) before library construction and sequencing.

### Transcriptome data analysis and identification of DEGs

The libraries for RNA-Seq were constructed according to the manufacturer’s standard protocol (Illumina, San Diego, USA) after total RNA quality control. mRNA was extracted from the total RNA of DC, RC, and AC meristems using Dynabeads bound with Oligo dT, followed by mRNA random fragmentation using fragmentation buffer. First- and second-strand cDNA were synthesized consecutively followed by cDNA purification. After end repair, dA addition to 3′-ends, adaptor ligation, and another purification, the cDNA library constructed by PCR amplification was sequenced with an Illumina HiSeq X-ten after quality control.

High-quality clean reads from the RNA-Seq were mapped to the *Populus trichocarpa* v3.0 reference genome (https://phytozome.jgi.doe.gov/pz/portal.html) to acquire their locations using TopHat2. Cuffquant and Cuffnorm were used to quantify the gene expression among the three stages from the RNA-Seq using FPKM values (fragments per kilobase of transcript per million fragments mapped). *K*-means analyses were conducted with standardized FPKM values to analyze the different expression patterns among the three stages. The DEGs were analyzed by DESeq with a fold change ≥ 4 and FDR < 0.01. A Venn diagram was used to demonstrate the numbers of specific and common DEGs among the three samples. Hierarchical clustering analysis of the DEGs was used to cluster the genes with the same or similar expression patterns. The functions of the DEGs were annotated by using GO enrichment analysis.

### sRNA-Seq and data analysis

The library for sRNA-Seq was constructed as described in the NEB Next Ultra Small RNA Sample Library Prep Kit for Illumina. Briefly, 1.5 μg of total RNA from dormant, reactivating, and active samples after quality control was used initially, followed by the addition of water to 6 μL. Fifteen percent denaturing polyacrylamide gel was used to isolate sRNA molecules ranging from 18 to 30 nt. After 5′- and 3′-end adaptor ligation with T4 RNA Ligase 1 and T4 RNA Ligase 2 (truncated), reverse transcription was conducted followed by PCR amplification. The amplification products were isolated by gel purification. The sRNA library was subjected to sequencing with an Illumina HiSeq X-ten (SE50).

All of the low-quality reads from the sRNA-Seq containing 3′-adapter reads, 5′-adapter contaminants, and sequences <18 nt or >30 nt were removed during data processing. Clean reads were BLASTed with Silva (https://www.arb-silva.de/), GtRNAdb (http://gtrnadb.ucsc.edu/), Repbase (http://www.girinst.org/repbase/), and Rfam (http://rfam.xfam.org/) to discard ncRNAs, including ribosomal RNA (rRNA), transfer RNA, small nuclear RNA, and small nucleolar RNA using Bowtie. All of the unannotated reads containing miRNAs were mapped to the reference genome (*P. trichocarpa v3.0*, https://phytozome.jgi.doe.gov/pz/portal.html). The unique sRNA and total sRNA were visualized with a Venn diagram. Mapped reads completely aligned to the mature sequence of miRbase (http://www.mirbase.org/) were identified as known miRNAs. miRDeep2 was used to predict novel miRNAs from the remaining unannotated reads. The abundance of miRNA was calculated based on transcripts per million values. DESeq was used to analyze differentially expressed miRNAs among three samples (|log2(FC) | ≥ 2; FDR ≤ 0.01). miRNAs with the same or similar expression patterns were clustered for hierarchical clustering analysis. According to the known and novel miRNAs and gene information of the corresponding species, TargetFinder was used to predict the target genes. Differentially expressed miRNAs were aligned by BLAST with the Nonredundant, Swiss-Prot, GO, KEGG, EuKaryotic Orthologous Groups, and Pfam databases to acquire annotation information for the target genes.

### qRT-PCR of candidate miRNAs and genes

Total RNA and miRNA were extracted from the vascular cambium of the three stages for qRT-PCR analysis to validate the expression levels of the DEGs. Reserve transcription of the miRNA was carried out with TransScript miRNA First-Strand cDNA Synthesis SuperMix (Trans, Beijing) following the supplied protocol. Reverse transcription was conducted with TransScript All-in-One First-Strand cDNA Synthesis SuperMix for qPCR (One-Step gDNA Removal) (Trans) according to the manufacturer’s instructions. Six miRNAs and 18 DEGs were selected. The primers of the miRNAs were designed using the mature sequences. The primers for the miRNAs and other DEGs are shown in the Supplementary Materials (Supplementary Table S11). All of the primers were designed using Primer Premier 6.0.

qRT-PCR was performed with TransStart Tip Green qPCR SuperMix (Trans) for miRNAs and TransStart Top Green qPCR SuperMix (Trans) according to the standard protocol on a Bioer 96plus (Bioer, Hanzhou). Three biological and technical replicates were performed. The procedure was as follows: qRT-PCRs were performed in 20 µL volumes containing 1 µL diluted cDNA, 250 nM of each primer, and 10 µL of Transtart Tip Green qPCR SuperMix or TransStart Top Green qPCR SuperMix. The amplification program was 94 °C for 30 s and 45 cycles at 94 °C for 5 s and 60 °C for 30 s. Relative expression levels of selected miRNAs were standardized to the expression levels of 5.8S rRNA. The relative expression levels of the miRNA target genes and other candidate DEGs were standardized with the expression levels of *PtrACTIN*. The 2^−ΔΔCt^ method was adopted to calculate the expression levels using the Ct value.

### Whole-genome bisulfite sequencing and data analysis

Library construction and sequencing for bisulfite sequencing were conducted according to the manufacturer’s standard protocol (Illumina, San Diego, USA). Genomic DNA was fragmented by ultrasonic treatment after quality qualification. The fragmented DNA was then treated with end repair, dA addition to the 3′-end, and methylated adaptor ligation. After isolation by agarose gel electrophoresis, 120–170 bp DNA was treated with bisulfite. A methylation library was constructed by PCR amplification of 16 cycles. Whole-genome bisulfite sequencing was conducted with an Illumina HiSeq X-ten according to the manufacturer’s protocol, following library quality-control standards.

Raw reads obtained from sequencing the vascular cambium of the three stages were evaluated and filtered to obtain clean reads for subsequent bioinformatics analysis. Clean reads were mapped to the reference genome (*P. trichocarpa v3.0*, https://phytozome.jgi.doe.gov/pz/portal.html). Alignment and methylation calling were performed according to the Bismark Bisulfite Mapper–User Guide v0.15.0 (http://www.bioinformatics.babraham.ac.uk/projects/bismark/Bismark_User_Guide.pdf). Methylation sites were detected using a binomial distribution test. The sites with a coverage depth >4 were filtered, followed by the generation of the coverage and total coverage of methylcytosine using Bismark_methylation_extractor. Benjamini and Hochberg FDR correction was used to identify the methylation sites (FDR < 0.05). The methylation sites of the three types (CG, CHG, and CHH) were calculated as described previously^74^.

The methylation distribution map at the chromosome level was created to describe the distribution of methylcytosine at each chromosome. To analyze the distribution of methylation sites on different regions of the poplar genome, the methylation level of the repeat region was detected with RepeatMasker (http://www.repeatmasker.org/). Model based analysis of bisulfite sequencing data was used to identify the DMRs among the three samples. A coverage depth ≥ 10, at least three differentially methylated sites, and Fisher’s exact test (*P* < 0.05) were required to identify differentially methylated sites. DMR gene annotation was performed according to the genome location of the DMRs and genome annotation. BLAST with GO and KEGG analyses was used to explore the biological function of the DMR genes.

## Supplementary information

Supplymentary Figures

Table S1

Table S2

Table S3

Table S4

Table S5

Table S6

Table S7

Table S8

Table S9

Table S10

Table S11

## Data Availability

All the original data (transcriptomes, small RNA sequencing, and DNA methylomes) used in this study have been deposited in the Genome Sequence Archive under the accession number CRA003637 and other data referred to are included in the article or Supplementary Materials.
