# Supplementary material for: Transcriptomic and epigenomic remodeling occurs during vascular cambium periodicity in Populus tomentosa
Source: Hortic Res. 2021 May 1;8:102. doi: 10.1038/s41438-021-00535-w (PMC8087784; doi:10.1038/s41438-021-00535-w)
Supplement: Supplementary file 1 — Supplymentary Figures [file 41438_2021_535_MOESM1_ESM.pdf]

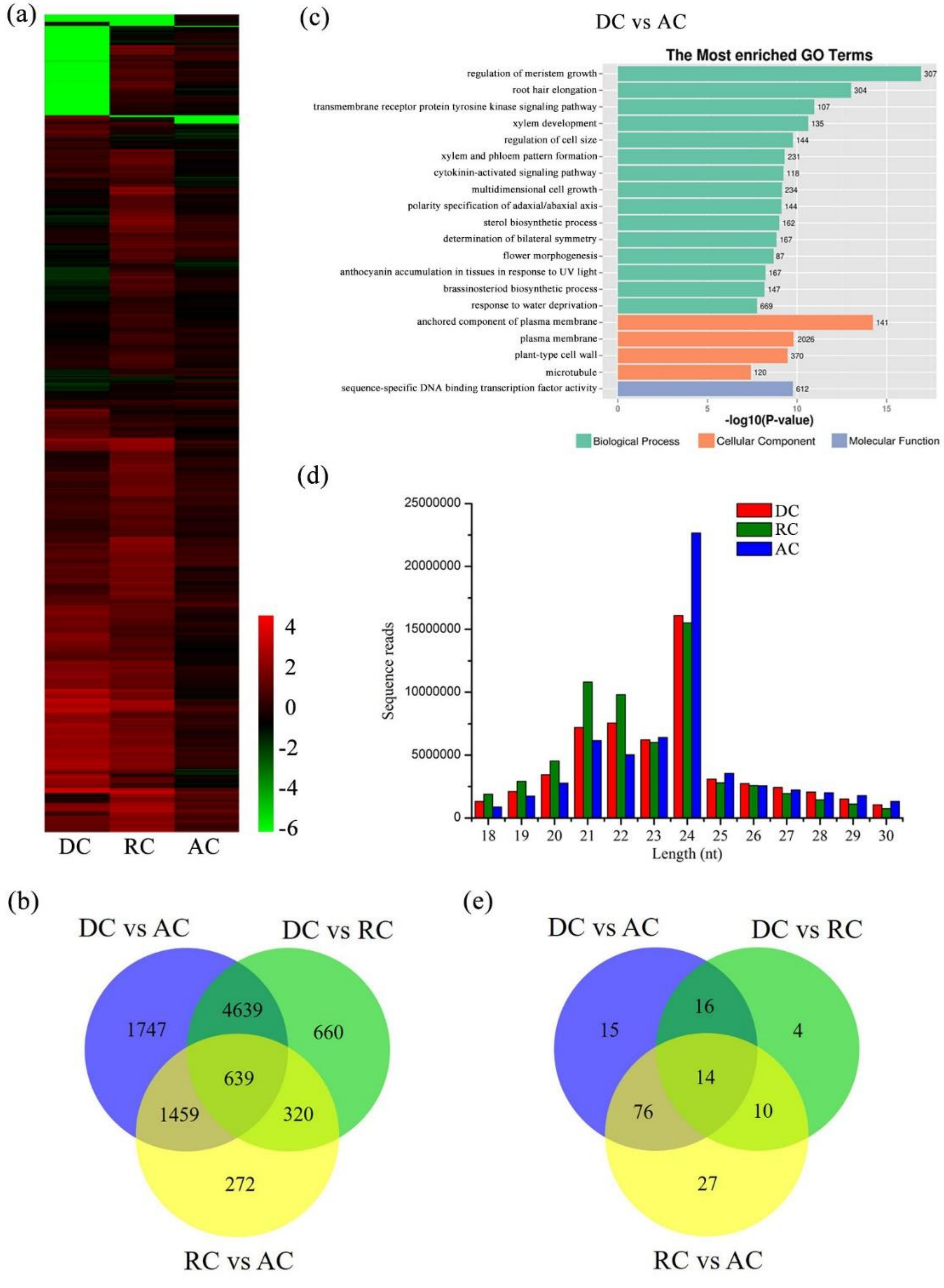
(a)
4
2
0
-2
-4
-6
DC
RC
AC
(c)
DC vs AC
The Most enriched GO Terms
regulation of meristem growth
root hair elongation
transmembrane receptor protein tyrosine kinase signaling pathway
xylem development
regulation of cell size
xylem and phloem pattern formation
cytokinin-activated signaling pathway
multidimensional cell growth
polarity specification of adaxial/abaxial axis
sterol biosynthetic process
determination of bilateral symmetry
flower morphogenesis
anthocyanin accumulation in tissues in response to UV light
brassinosteriod biosynthetic process
response to water deprivation
anchored component of plasma membrane
plasma membrane
plant-type cell wall
microtubule
sequence-specific DNA binding transcription factor activity
307
304
107
135
144
231
118
234
144
162
167
87
167
147
669
141
2026
370
120
612
0
5
10
15
-log10(P-value)
Biological Process
Cellular Component
Molecular Function
(d)
25000000
20000000
15000000
10000000
5000000
0
Sequence reads
DC
RC
AC
18
19
20
21
22
23
24
25
26
27
28
29
30
Length (nt)
(b)
DC vs AC
DC vs RC
1747
4639
660
639
1459
320
272
RC vs AC
(e)
DC vs AC
DC vs RC
15
16
4
14
76
10
27
RC vs AC

**Fig. S1 Overview of the extensive transcriptomic analysis and miRNA profiling during the cambium activity periodicity.** (a) Heatmap showing the expression patterns of all DEGs during cambium development. (b) Venn diagram of the number of DEGs during pairwise comparisons among dormant, reactivating, and active cambium. (c) GO analysis of DEGs from DC vs. AC. (d) Histogram showing the length distribution of the sRNAs identified from the cambium of the three stages in poplar. (e) Venn diagram of the number of differentially expressed miRNAs during pairwise comparisons among dormant, reactivating, and active cambium. DEGs: differentially expressed genes. DC: dormant cambium, RC: reactivating cambium, AC: active cambium, nt: nucleotides.

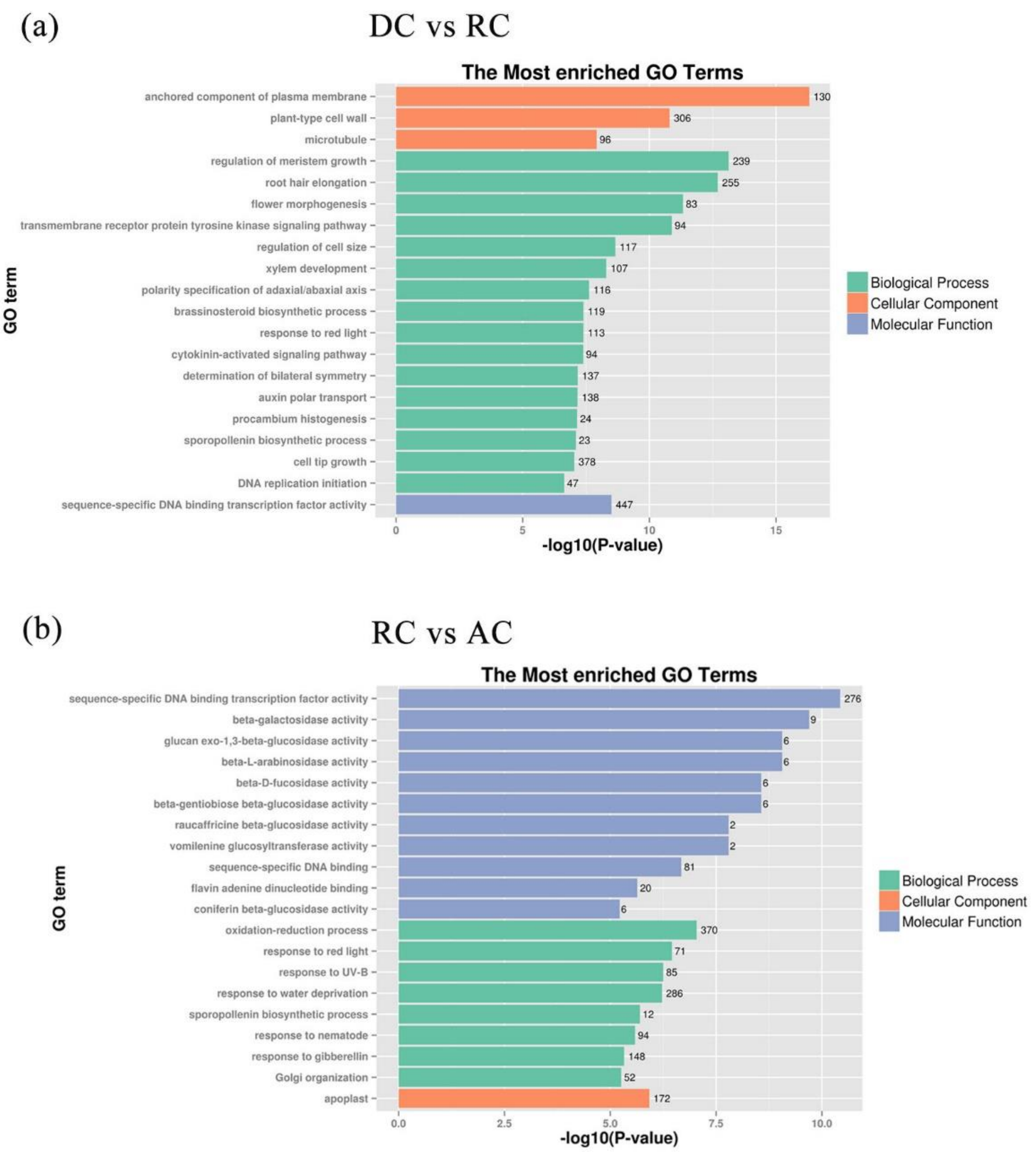


**Fig. S2 GO enrichment analysis of DEGs from DC vs. RC and RC vs. AC.** GO terms are categorized into three groups: biological process, cellular component, and molecular function. DEGs: differentially expressed genes. DC: dormant cambium, RC: reactivating cambium, AC: active cambium.

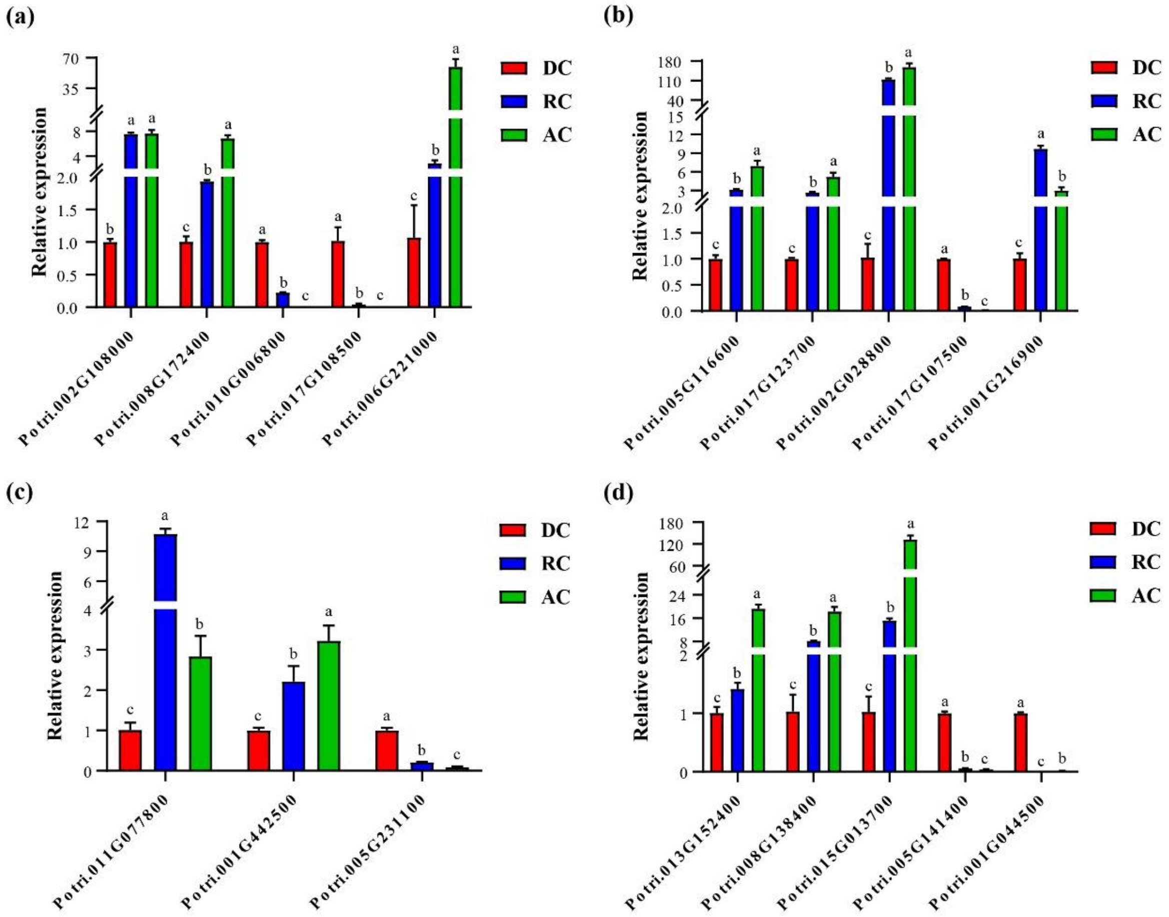


**Fig. S3 qRT-PCR analysis of the expression of 18 vascular developmental-associated DEGs in *Populus tomentosa* cambium during cambium activity periodicity.** (a) Expression patterns of five genes associated with plant hormone transduction; Potri.002G108000, Potri.008G172400, Potri.010G006800, Potri.017G108500, Potri.006G221000 encoding auxin-responsive family protein, IAA13-like protein, AP2 domain transcription factor family protein, ABA-inducible family protein, and Cytokinin oxidase family protein, respectively. (b) Expression patterns of five genes involved in cell division; Potri.005G116600, Potri.017G123700, Potri.002G028800, Potri.017G107500, Potri.001G216900 encoding Histone H1, H2B, H3, COP1-interactive protein 1 and Transcription factor LHW-like, respectively. (c) Expression patterns of three genes involved in cellular transport. Potri.011G077800, Potri.001G442500, Potri.005G231100 encoding AWPM-19-like membrane family protein, Vacuolar protein sorting-associated protein 2 and MAP kinase PsMAPK2

family protein, respectively. (d) Expression patterns of five genes involved in cell wall biosynthesis. Potri.013G152400, Potri.008G138400, Potri.015G013700, Potri.005G141400, Potri.001G044500 encoding xyloglucan endotransglucosylase/ hydrolase protein 9 precursor, xyloglucan endotransglycosylase family protein, pectin methylesterase family protein, WRKY transcription factor 15 family protein and WRKY transcription factor 40 family protein, respectively. Experiments were performed with three biological replicates × three technical replicates. Significant differences are denoted by different letters ($P < 0.05$; one-way ANOVA followed by a Tukey's multiple range test). DC: dormant cambium; RC, reactivating cambium; AC: active cambium.

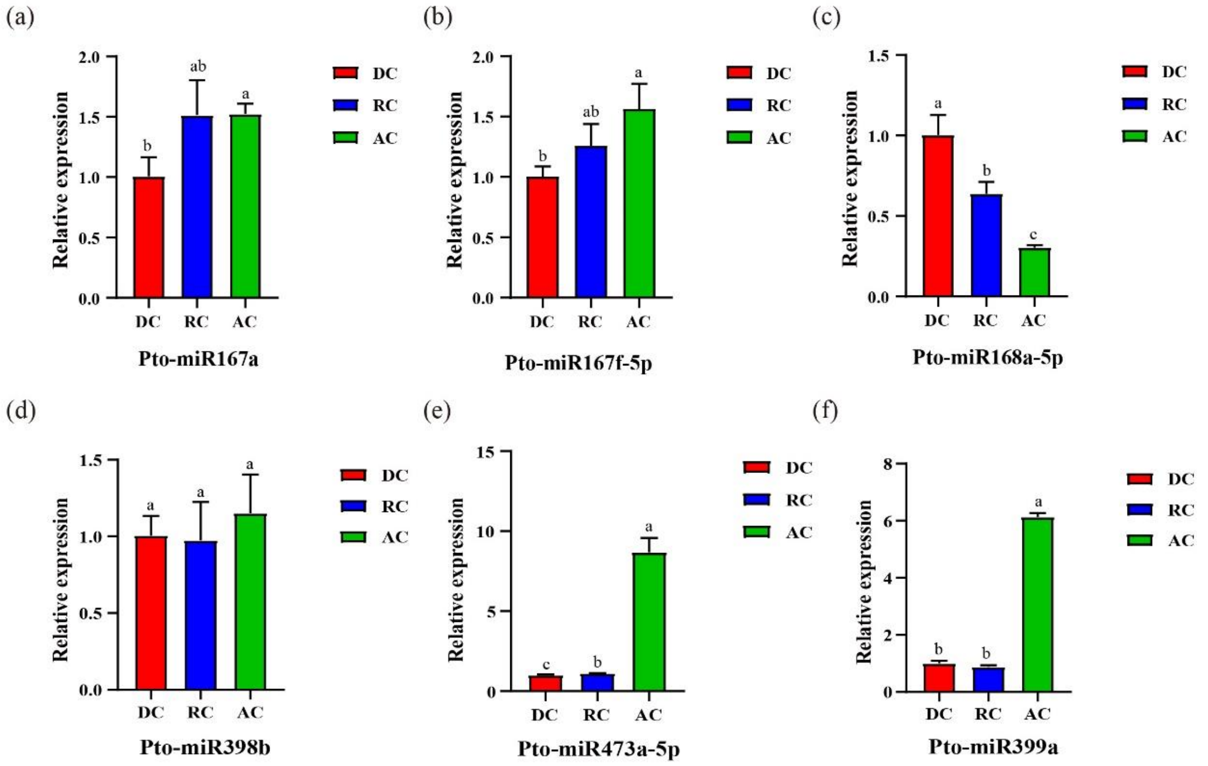


**Fig. S4 qRT-PCR analysis of the expression of six random differentially expressed miRNAs in *Populus tomentosa* cambium during cambium activity periodicity.** Histogram showing the expression patterns of Pto-miR167a (a), Pto-miR167f-5p (b), Pto-miR168a-5p (c), Pto-miR398b (d), Pto-miR473a-5p (e), and Pto-miR168a-5p (f). Experiments were carried out with three biological replicates × three technical replicates. Significant differences are denoted by different letters ($P < 0.05$; one-way ANOVA followed by a Tukey's multiple range test). DC: dormant cambium; RC, reactivating cambium; AC: active cambium.

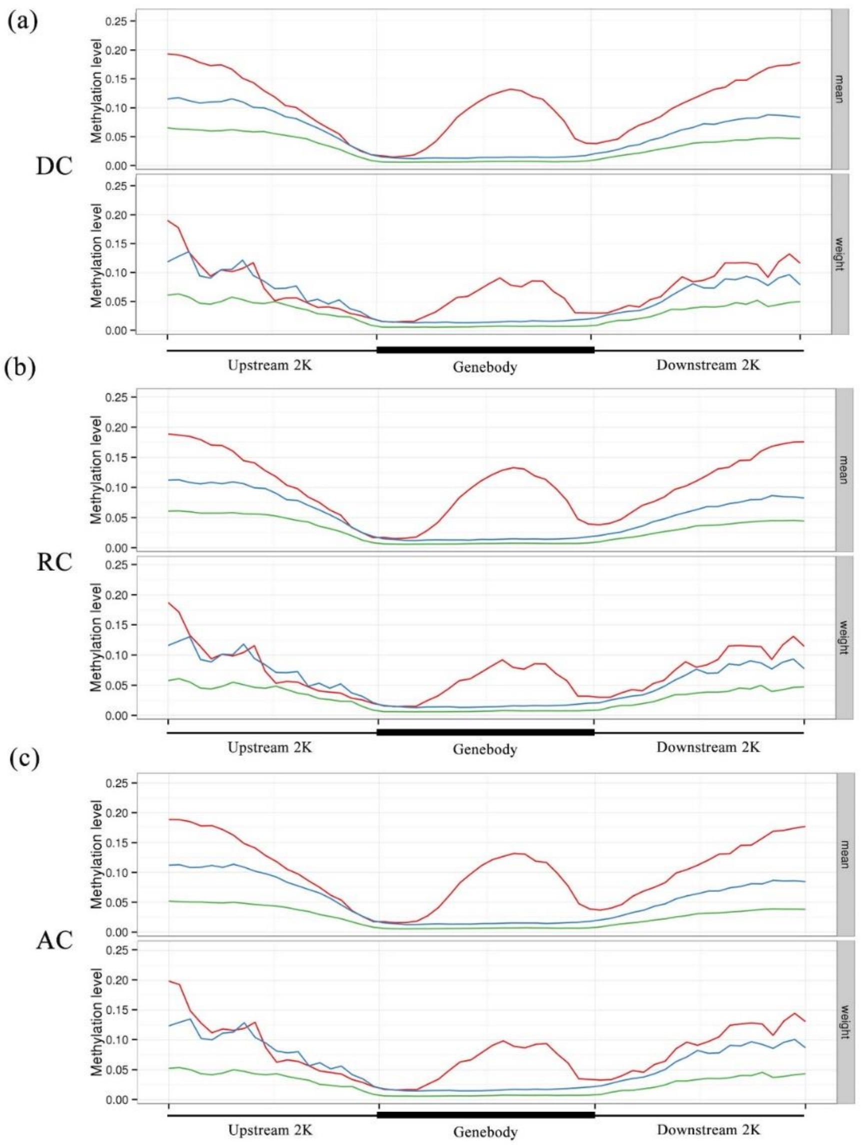


**Fig. S5 DNA methylation patterns counted using the methods of mean and weight ways.** DNA methylation patterns of DC, RC, and AC in different genomic regions for each methylation context were counted using the methods of mean and weight ways. DC: dormant cambium, RC: reactivating cambium, AC: active cambium.

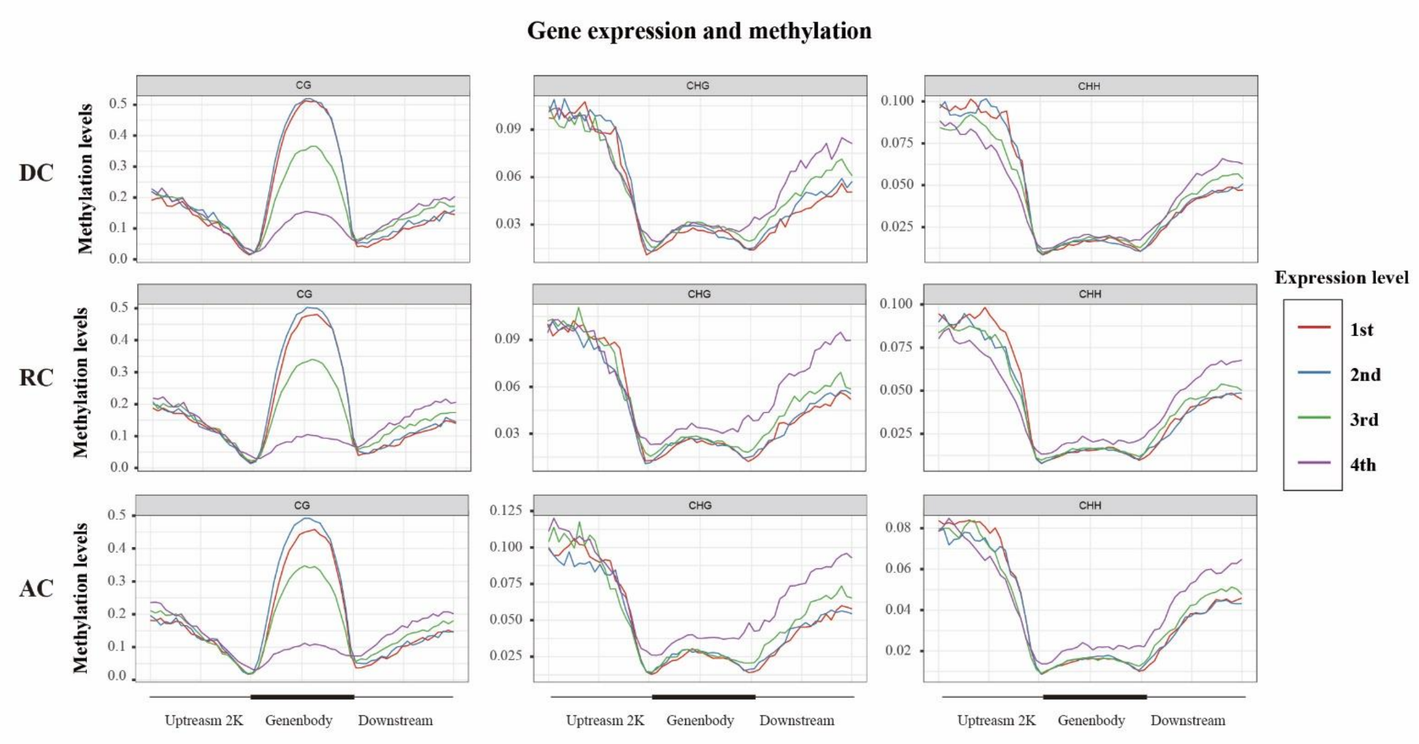


**Fig. S6 Correlation between DNA methylation and gene expression.** Distributions of methylation levels of expressed genes, which were divided into quintiles based on promoter, gene body, and downstream 2-kb region. Expression level: 1st is the highest and 4th is the lowest. DC: dormant cambium; RC, reactivating cambium; AC: active cambium.

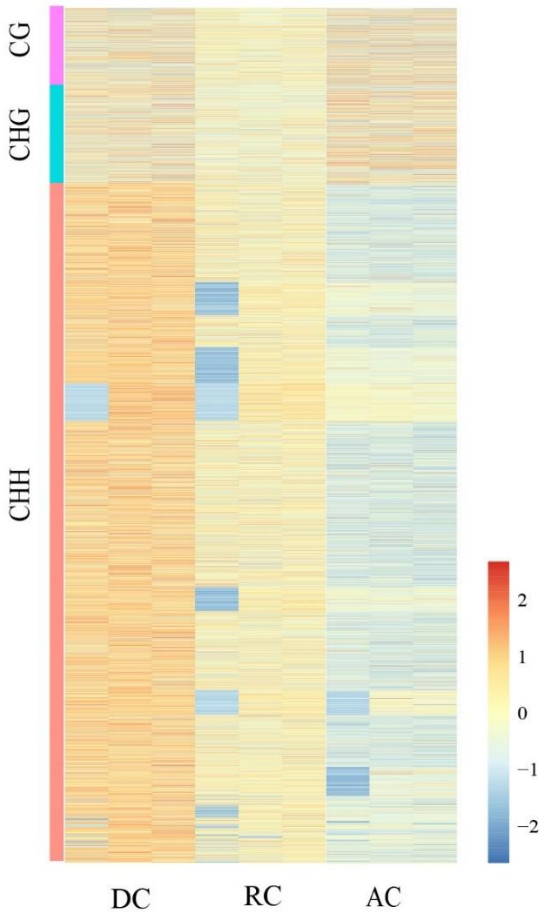


**Fig. S7 Patterns of CG, CHG, and CHH DMR during cambium periodicity.** Heatmaps showing the methylation levels in each methylation context and each sample. Red represents a high methylation level and green represents low methylation level. DC: dormant cambium, RC: reactivating cambium, AC: active cambium.

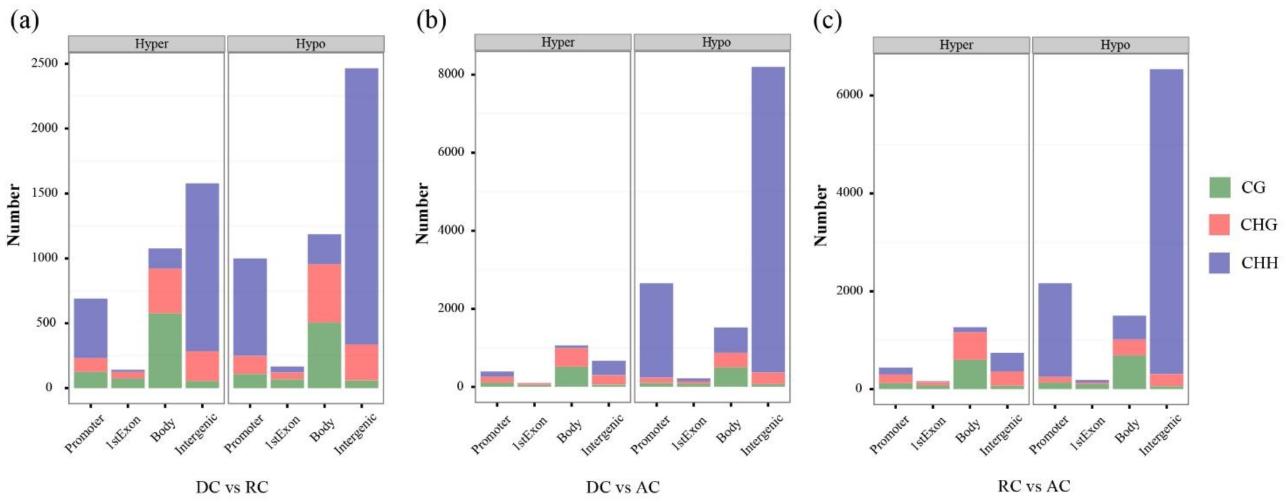


**Fig. S8 Analysis of the location and annotation for DMRs by pairwise comparison.** Histogram showing the location and annotation of DMRs in different genomic regions during the comparison of DC vs RC, DC vs AC, and RC vs AC, respectively. DC: dormant cambium, RC: reactivating cambium, AC: active cambium.

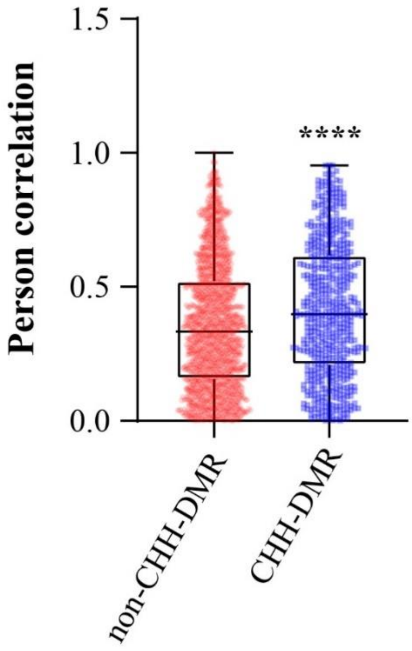


**Fig. S9 The expression correlation with the DNA methylation levels of CHH-DMR overlapped with gene promoter and non-CHH-DMR overlapped genes.** The expression correlation was calculated by Person correlation coefficient. Significant differences are denoted by asterisks ($^{****}P$<0.0001; Student's *t* test).

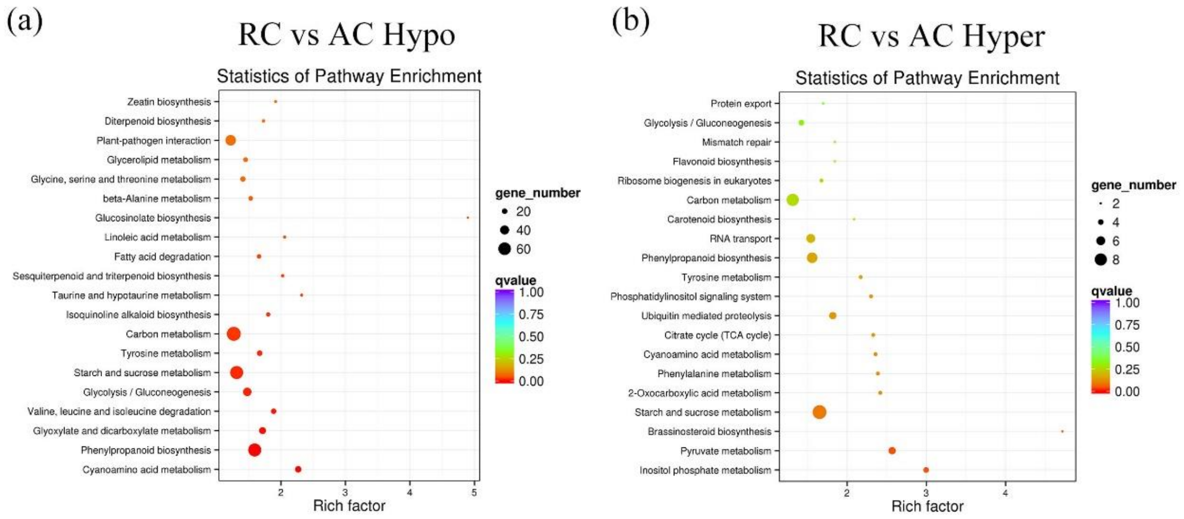


**Fig. S10 Functional annotation of hyper and hypomethylated genes from RC vs. AC.** KEGG analysis of hypermethylated and hypomethylated genes from RC vs. AC. RC: reactivating cambium, AC: active cambium.

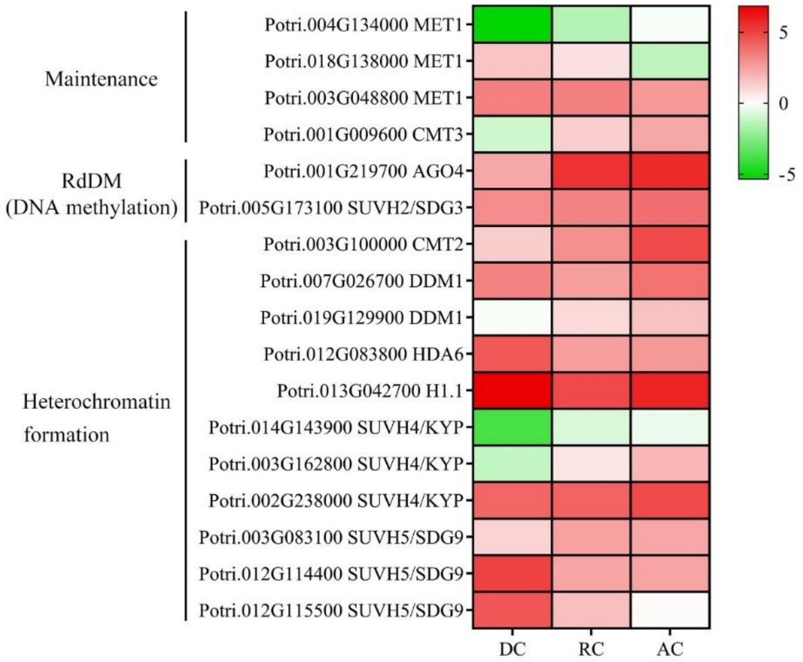


**Fig. S11 Expression patterns of genes associated with different DNA methylation pathways.** DC: dormant cambium; RC, reactivating cambium; AC: active cambium.
