# Supplementary material for: Transcriptomic and epigenomic remodeling occurs during vascular cambium periodicity in Populus tomentosa
Source: Hortic Res. 2021 May 1;8:102. doi: 10.1038/s41438-021-00535-w (PMC8087784; doi:10.1038/s41438-021-00535-w)
Supplement: Supplementary file 2 — Table S1 [file 41438_2021_535_MOESM2_ESM.docx]

**Table S1 Summary for transcriptome from different stages of vascular cambium in *Populus tomentosa.***

| **Samples** | **Clean reads** | **Clean bases** | **GC contents** | **%>Q30** |
| --- | --- | --- | --- | --- |
| DC1 | 26,425,978 | 7,882,830,416 | 44.51% | 90.52% |
| DC2 | 25,643,972 | 7,648,148,912 | 44.37% | 90.80% |
| DC3 | 20,541,062 | 6,119,826,770 | 44.39% | 90.95% |
| RC1 | 20,387,450 | 6,091,320,110 | 44.98% | 90.84% |
| RC2 | 21,523,874 | 6,421,574,728 | 45.48% | 90.51% |
| RC3 | 20,680,978 | 6,174,157,870 | 45.66% | 90.86% |
| AC1 | 27,257,507 | 8,148,780,192 | 44.24% | 90.92% |
| AC2 | 23,597,901 | 7,058,659,910 | 44.09% | 90.27% |
| AC3 | 20,543,500 | 6,142,770,558 | 44.33% | 90.22% |

Note: DC, dormant cambium; RC, reactivating cambium; AC, active cambium.
