# Supplementary material for: Transcriptomic and epigenomic remodeling occurs during vascular cambium periodicity in Populus tomentosa
Source: Hortic Res. 2021 May 1;8:102. doi: 10.1038/s41438-021-00535-w (PMC8087784; doi:10.1038/s41438-021-00535-w)
Supplement: Supplementary file 5 — Table S4 [file 41438_2021_535_MOESM5_ESM.docx]

**Table S4 The length distribution from small RNA libraries of different vascular cambium samples in *Populus tomentosa.***

| **Length (nt)** | **DC** | **RC** | **AC** |
| --- | --- | --- | --- |
| 18 | 1326125 | 1891990 | 878475 |
| 19 | 2103575 | 2921202 | 1735967 |
| 20 | 3449656 | 4526686 | 2773858 |
| 21 | 7207420 | 10808883 | 6149343 |
| 22 | 7552664 | 9808206 | 5018242 |
| 23 | 6219741 | 6013384 | 6405305 |
| 24 | 16100188 | 15515490 | 22652539 |
| 25 | 3095417 | 2799362 | 3540293 |
| 26 | 2734635 | 2570651 | 2556276 |
| 27 | 2431849 | 1949963 | 2219640 |
| 28 | 2068113 | 1443376 | 1997095 |
| 29 | 1501939 | 1108987 | 1775639 |
| 30 | 1058831 | 747219 | 1325105 |

Note: DC, dormant cambium; RC, reactivating cambium; AC, active cambium. The number represents the average count of clean reads.
