# Supplementary material for: Transcriptomic and epigenomic remodeling occurs during vascular cambium periodicity in Populus tomentosa
Source: Hortic Res. 2021 May 1;8:102. doi: 10.1038/s41438-021-00535-w (PMC8087784; doi:10.1038/s41438-021-00535-w)
Supplement: Supplementary file 9 — Table S8 [file 41438_2021_535_MOESM9_ESM.docx]

**Table S8 Summary for DNA methylome and conversion rate from different stages of vascular cambium in *Populus tomentosa.***

| **Samples** | **Clean Base** | **Clean Reads** | **Unique mapped Reads** | **Total Cytosine Site** | **Total Converted Cytosine Site** | **Bisulfite Conversion**  **Rate (%)** |
| --- | --- | --- | --- | --- | --- | --- |
| DC1 | 11864032298 | 39584187 | 12836641 | 6723948 | 6700368 | 99.65 |
| DC2 | 12420273552 | 41506448 | 13681351 | 6358866 | 6335716 | 99.64 |
| DC3 | 10532273528 | 35180209 | 11337925 | 6285348 | 6263194 | 99.65 |
| RC1 | 10878519628 | 36298083 | 11482920 | 8292947 | 8261603 | 99.62 |
| RC2 | 12095583622 | 40364382 | 12976878 | 6952053 | 6931454 | 99.70 |
| RC3 | 12385359758 | 41331905 | 13268037 | 9388020 | 9352042 | 99.62 |
| AC1 | 13561805426 | 45262171 | 14218224 | 6590334 | 6561779 | 99.57 |
| AC2 | 12079894452 | 40308286 | 12895693 | 6120108 | 6099239 | 99.66 |
| AC3 | 10494971620 | 35016959 | 10834393 | 6677679 | 6651131 | 99.60 |

Note: DC, dormant cambium; RC, reactivating cambium; AC, active cambium.
