# Supplementary material for: Transcriptomic and epigenomic remodeling occurs during vascular cambium periodicity in Populus tomentosa
Source: Hortic Res. 2021 May 1;8:102. doi: 10.1038/s41438-021-00535-w (PMC8087784; doi:10.1038/s41438-021-00535-w)
Supplement: Supplementary file 10 — Table S9 [file 41438_2021_535_MOESM10_ESM.docx]

**Table S9 The number of methylcytosine sites in different contexts from the dormant, reactivating, and active cambium in *Populus tomentosa.***

| **Sample** | **CG(+)** | **CHG(+)** | **CHH(+)** | **CG(-)** | **CHG(-)** | **CHH(-)** | **CG(%)** | **CHG(%)** | **CHH(%)** |
| --- | --- | --- | --- | --- | --- | --- | --- | --- | --- |
| DC1 | 542122 | 489968 | 1742988 | 468507 | 400330 | 1413781 | 19.98 | 17.6 | 62.42 |
| DC2 | 561288 | 522248 | 1895096 | 486006 | 430403 | 1555095 | 19.22 | 17.48 | 63.3 |
| DC | 499480 | 458645 | 1616661 | 428019 | 375562 | 1316054 | 19.76 | 17.77 | 62.47 |
| RC1 | 517392 | 464220 | 1611963 | 444383 | 377113 | 1305672 | 20.37 | 17.82 | 61.8 |
| RC2 | 531242 | 471131 | 1655322 | 459437 | 385796 | 1344402 | 20.44 | 17.68 | 61.88 |
| RC3 | 556041 | 511854 | 1835102 | 481751 | 422354 | 1502840 | 19.54 | 17.59 | 62.86 |
| AC1 | 585356 | 554204 | 1807866 | 509009 | 458966 | 1487599 | 20.25 | 18.75 | 60.99 |
| AC2 | 545415 | 495142 | 1555934 | 472598 | 404942 | 1262186 | 21.49 | 19 | 59.5 |
| AC3 | 503311 | 455526 | 1405235 | 432677 | 371568 | 1131694 | 21.77 | 19.23 | 59 |

Note: Filter condition for methylcytosine site: coverage > = 4X and FDR<0.05; DC, dormant cambium; RC, reactivating cambium; AC, active cambium.
