# Supplementary material for: Transcriptomic and epigenomic remodeling occurs during vascular cambium periodicity in Populus tomentosa
Source: Hortic Res. 2021 May 1;8:102. doi: 10.1038/s41438-021-00535-w (PMC8087784; doi:10.1038/s41438-021-00535-w)
Supplement: Supplementary file 11 — Table S10 [file 41438_2021_535_MOESM11_ESM.docx]

**Table S10 The location and annotation of DMRs in different genomic regions by comparison among the dormant, reactivating, and active cambium in *Populus tomentosa.***

|  | **Hyper** | | |  | **Hypo** | | |
| --- | --- | --- | --- | --- | --- | --- | --- |
|  | **CG** | **CHG** | **CHH** |  | **CG** | **CHG** | **CHH** |
| **DC vs AC** |  |  |  |  |  |  |  |
| Promoter | 109 | 150 | 136 |  | 105 | 130 | 2423 |
| 1st Exon | 54 | 44 | 6 |  | 80 | 54 | 88 |
| Body | 515 | 485 | 64 |  | 502 | 371 | 650 |
| Intergenic | 63 | 242 | 366 |  | 70 | 302 | 7827 |
| **DC vs RC** |  |  |  |  |  |  |  |
| Promoter | 126 | 107 | 457 |  | 110 | 139 | 751 |
| 1st Exon | 75 | 50 | 17 |  | 68 | 54 | 45 |
| Body | 577 | 345 | 155 |  | 507 | 449 | 231 |
| Intergenic | 56 | 227 | 1296 |  | 59 | 278 | 2129 |
| **RC vs AC** |  |  |  |  |  |  |  |
| Promoter | 118 | 178 | 145 |  | 135 | 118 | 1911 |
| 1st Exon | 76 | 72 | 15 |  | 98 | 33 | 52 |
| Body | 596 | 572 | 98 |  | 686 | 327 | 489 |
| Intergenic | 67 | 292 | 380 |  | 63 | 248 | 6230 |

Note: DMRs, different methylation regions; DC, dormant cambium; RC, reactivating cambium; AC, active cambium.
