# Supplementary material for: Transcriptomic and epigenomic remodeling occurs during vascular cambium periodicity in Populus tomentosa
Source: Hortic Res. 2021 May 1;8:102. doi: 10.1038/s41438-021-00535-w (PMC8087784; doi:10.1038/s41438-021-00535-w)
Supplement: Supplementary file 12 — Table S11 [file 41438_2021_535_MOESM12_ESM.docx]

**Table S11 Primer sequences used for qRT-PCR.**

| **Gene Name** | **Forward primers** | **Reverse primers** |
| --- | --- | --- |
| **For DEGs** |  |  |
| Potri.002G108000  Potri.008G172400  Potri.010G006800  Potri.017G108500  Potri.006G221000  Potri.005G116600  Potri.017G123700  Potri.002G028800  Potri.017G107500  Potri.001G216900  Potri.011G077800  Potri.001G442500  Potri.005G231100  Potri.013G152400  Potri.008G138400  Potri.015G013700  Potri.005G141400  Potri.001G044500 | ACTTCGTAACAGCTCCGCAA  CCAGCCTCAAAGGCCTAACA  TCCTGAAACGGCTGCTATGG  GGCCAGTACCTTGGTGAACA  GGGGAAACCTTCGTCGATGT  AACTCTGCAGCGAGAGGAAA  TGGGATTTCAAGCAAGGCGA  CTTCCCTTCCAAAGGCTCGT  GGGATTGGAGTATGAGCCCG  GCTGGAGAGAACCCAAGACC  TCGGACCTGGGTATGACCTT  GTGCGACTTCGGCAACAAAT  TCGTTGGTATCGTGCTCCAG  TTCGTCGCCTCCTACAAAGG  CCTTGTTCTGCACGGTTGTG  TGTTCATCCAAGAGGGTGGC  TTCTCGGGTTTCTTCAGCGG  CTCGGTTCAGTACCCTGCTC | CTGGCCCTGCTTTACCATCA  ATCCACCCTCATGGGAGACA  TCAAGCATCCAAGGGTCCAC  TGTGCCGTAGACATCACCTG  TGCTACGTTGCACGTTTGTG  TCACTGCACTACTTCCCTTGG  ACAGCAGTCTGGATCTCCCT  GACCCACGAGATAAGCCTCG  CGCTCATTGCTGATTCTGGC  CTGCATGCTTTGTCACGCTT  AACAATTGCCGAGGCAACAC  GCTTGTTTTGCTGGAGCCAT  TGAGACATTCTGTGCCAGGG  ATGGCTCTGGTGCACATTGA  GAACCTTGTACCGAACCCGA  GCCATTTAACCCTTTGCCCG  GATTGCTGGAACCCTCACCA  GGCCATCTGTTCCACCAAGA |
| **For miRNAs** |  |  |
| Ptc-miR167a  Ptc-miR167f-5p  Ptc-miR168a-5p  Ptc-miR398b  Ptc-miR399a  Ptc-miR473a-5p | CTGAAGCTGCCAGCATGATC  TGAAGCTGCCAGCATGATC  TCGCTTGGTGCAGGTCGGGA  AGCCTGTGTTCTCAGGTCGC  GGCTGCCAAAGGAGATTTGC  ACTCTCCCTCAAGGCTTCCA | Universal miRNA qPCR Primer  Universal miRNA qPCR Primer  Universal miRNA qPCR Primer  Universal miRNA qPCR Primer  Universal miRNA qPCR Primer  Universal miRNA qPCR Primer |
